# Identification of *Schistosoma haematobium* and *Schistosoma mansoni* linear B-cell epitopes with diagnostic potential using *in silico* immunoinformatic tools and peptide microarray technology

**DOI:** 10.1371/journal.pntd.0011887

**Published:** 2024-08-22

**Authors:** Arthur Vengesai, Marble Manuwa, Herald Midzi, Masimba Mandeya, Victor Muleya, Keith Mujeni, Isaac Chipako, Takafira Mduluza

**Affiliations:** 1 Department of Biochemistry, Faculty of Medicine and Health Sciences, Midlands State University, Senga Road, Gweru, Zimbabwe; 2 Department of Biotechnology and Biochemistry, Faculty of Science, University of Zimbabwe, Mount Pleasant, Harare, Zimbabwe; 3 Department of Applied Biosciences and Biotechnology, Faculty of Science, Midlands State University, Senga Road, Gweru, Zimbabwe; 4 Partnership in Education Training and Research Advancement, Faculty of Health Sciences, University of Zimbabwe, Harare, Zimbabwe; 5 Health Economics and Policy Department, Division of Health Research Graduate College, Lancaster University, Lancaster, United Kingdom; Centers for Disease Control and Prevention, UNITED STATES OF AMERICA

## Abstract

**Introduction:**

Immunoinformatic tools can be used to predict schistosome-specific B-cell epitopes with little sequence identity to human proteins and antigens other than the target. This study reports an approach for identifying schistosome peptides mimicking linear B-cell epitopes using *in-silico* tools and peptide microarray immunoassay validation.

**Method:**

Firstly, a comprehensive literature search was conducted to obtain published schistosome-specific peptides and recombinant proteins with the best overall diagnostic performances. For novel peptides, linear B-cell epitopes were predicted from target recombinant proteins using ABCpred, Bcepred and BepiPred 2.0 *in-silico* tools. Together with the published peptides, predicted peptides with the highest probability of being B-cell epitopes and the lowest sequence identity with proteins from human and other pathogens were selected. Antibodies against the peptides were measured in sera, using peptide microarray immunoassays. Area under the ROC curve was calculated to assess the overall diagnostic performances of the peptides.

**Results:**

Peptide AA81008-19-30 had excellent and acceptable diagnostic performances for discriminating *S*. *mansoni* and *S*. *haematobium* positives from healthy controls, with AUC values of 0.8043 and 0.7326 respectively for IgG. Peptides MS3_10186-123-131, MS3_10385-339-354, SmSPI-177-193, SmSPI-379-388, MS3-10186-40-49 and SmS-197-214 had acceptable diagnostic performances for discriminating *S*. *mansoni* positives from healthy controls with AUC values ranging from 0.7098 to 0.7763 for IgG. Peptides SmSPI-359-372, Smp126160-438-452 and MS3 10186-25-41 had acceptable diagnostic performances for discriminating *S*. *mansoni* positives from *S*. *mansoni* negatives with AUC values of 0.7124, 0.7156 and 0.7115 respectively for IgG. Peptide MS3-10186-40-49 had an acceptable diagnostic performance for discriminating *S*. *mansoni* positives from healthy controls, with an AUC value of 0.7413 for IgM.

**Conclusion:**

One peptide with a good diagnostic performance and nine peptides with acceptable diagnostic performances were identified using the immunoinformatic approach and peptide microarray validation. There is need for evaluation of the peptides with true negatives and a good standard positive reference.

## Author summary

Schistosomiasis, commonly known as bilharzia, is the third most significant tropical disease after malaria and soil-transmitted helminthiases. Like other neglected tropical diseases common in Zimbabwe, schistosomiasis remains mostly undiagnosed or undetected. This is partly due to the fact that reliable identification of parasites requires expertise for specimen preparation, and microscopic examination. Unfortunately, this level of expertise is unavailable in most rural clinics. This limitation is further compounded by the fact that the recommended microscopy-based methods for schistosomiasis diagnosis lack sensitivity, especially in infections of low intensity. To overcome some of the problems associated with microscopy-based methods, highly sensitive serological tests have been utilized. Unfortunately, currently available serological tests have low specificity and show cross-reactivity with other helminthic infections. One way to mitigate cross-reactivity and increase the specificity, is to use immunoinformatic tools and immunoassays to identify schistosomiasis species-specific immunogenic peptides mimicking B-cell epitopes (short amino acid sequences of the antigen that reacts with antibodies). Utilizing immunoinformatic tools coupled with peptide microarray immunoassay validation approach several peptides that can be used to develop diagnostic tools for showing exposure to infection for people living in non-endemic or low-transmission areas were identified in the current study.

## 1 Introduction

Schistosomiasis is a neglected tropical disease caused by blood flukes from the genus *Schistosoma* [1]. Zimbabwe is endemic to urogenital schistosomiasis caused by *Schistosoma haematobium* and intestinal schistosomiasis caused by *Schistosoma mansoni* [2–4]. Besides effective implementation of mass drug administration (MDA) campaigns, access to safe water, improved sanitation and snail control, diagnostic tests are important tools for achieving and sustaining schistosomiasis elimination [1,5,6]. Diagnostic tests are important for schistosomiasis surveillance and control. They play a vital role in guiding the distribution of current program resources and the implementation and evaluation of schistosomiasis intervention strategies [5,6].

The recommended method for schistosomiasis diagnosis is the detection of schistosome-specific eggs in stool or urine specimens by microscopy. For urogenital schistosomiasis, the urine filtration technique is the standard diagnostic method and for intestinal schistosomiasis, the Kato-Katz technique is the standard diagnostic method [6–8]. In addition to high specificity [7,9], both techniques have minimal operational costs (the test kits are inexpensive), low complexity and both tests are relatively easy to perform in resource-limited field settings [6,8,9]. Moreover, prepared Kato-Katz slides can be stored for months at room temperature for later microscopic examination.

Nevertheless, both techniques have significant disadvantages including, the need for qualified personnel to prepare and examine slides, poor reproducibility and most importantly, low sensitivity [7,8,10,11]. The sensitivity of the techniques is limited by the host infection intensity, daily variation of schistosome egg excretion and uneven distribution of eggs within stool specimens [11]. Additionally, both methods are unable to diagnose recent infections (for instance, in cases where worms have not yet started to produce eggs) single-sex and non-fecund worm infections. The Kato-Katz technique is also unable to analyse watery stool specimens [9]. The lack of sensitivity especially in low endemic areas and after successful control interventions lead to the underestimation of true schistosomiasis prevalence in such settings [7–10]. Moreover, undetected and untreated individuals may continue schistosomiasis transmission by contaminating fresh water sources with urine and faeces containing schistosome eggs [1,9]. Due to the numerous disadvantages associated with the microscope based techniques, the use of other methods like molecular detection, circulating cathodic antigen (CCA), circulating anodic antigen (CAA) has gathered pace [8]. These alternative methods for schistosomiasis diagnosis have their own advantages and limitations too.

Molecular methods targeting DNA of the parasite are more sensitive and specific and can detect early stage schistosome infections [8]. However, molecular methods require skilled laboratory personnel, expensive and fragile equipment and they are time consuming, thus impeding their use as point-of-care tools (POC) in remote resource-limited endemic areas [5]. Although CCA and CAA allow for rapid schistosomiasis diagnosis, these methods have their own shortcomings [3,7,8]. The CCA test is sensitive for moderate to high level *S*. *mansoni* infections but not for *S*. *haematobium* infections, and its widespread use in poor rural endemic areas may be limited by its cost, currently around US $1.75 per test [3,12,13]. While the CAA test is more sensitive than the CCA, it is labour intensive and riddled with a complicated assay procedure. This major drawback is further compounded by the fact that there has not been any commercially available CAA tests to date [8].

To overcome some of the drawbacks associated with microscopy; molecular, CCA and CAA based diagnostic tests serological tests have been utilized [14]. The use of serological tests has gained traction due to higher sensitivities compared to microscopy based techniques [8]. However, currently available serological tests exhibit cross-reactivity with other helminthic infections due to shared antigenic epitopes [14,15]. Additional crude antigens and recombinant are of limited application in poor resource regions due to high costs of production (List 2010). One way to mitigate the cross-reactivity and increase specificity is to use bioinformatic and proteomics tools to predict schistosome specific immunodominant B-cell epitopes with little or no sequence identity to proteins other than the target [4,14,16]. However, to date, only a limited number of linear B-cell epitopes have been identified for the serological diagnosis of *S*. *haematobium* and *S*. *mansoni* [17–19]. It is against this background that we present an approach for predicting schistosome specific peptides mimicking linear B-cell epitopes using *in-silico* tools and peptide microarray technology. Several methods can be used for the identification and prediction of linear B-cell epitopes. As previously described, two methods *in-silico* prediction and identification of published peptides reduce the burden and costs associated with epitope mapping by decreasing the list of potential targets for experimental testing [20–24]. Therefore, in the present study a comprehensive literature search was conducted to identify published schistosome-specific peptides for inclusion in the peptide microarray immunoassays.

## 2 Methods

### 2.1. Ethical approval

Approval to conduct the study was obtained from Medical Research Council of Zimbabwe (MRCZ/A/2571). Provincial Medical directors, District Medical officers, councillors, and headmen provided the permission to carry out the study in the districts. Before enrolment, the aims and procedures of the study were explained to all participants and their parents or guardians in English or Shona. Adults were included into the study after signing informed consent forms. Children aged 1–11 years were enrolled into the study after their verbal assent and/or submission of signed consent forms by their parents or legal guardians. Children aged 12–18 years were recruited into the study after signing assent forms and submitting consent forms signed by their parents or legal guardians. Enrolment into the study was completely voluntary and parent or guardians were free to withdraw their children at any time with no need of explanation.

### 2.2. Study design, area and population

Village health workers and environmental health technicians who resided in the same communities and were familiar with the study participants were engaged in the research and involved in the recruitment of participants for inclusion in the cross-sectional study. Urine and stool specimens for the diagnosis of schistosomiasis, and sera for the peptide microarray immunoassays were collected at local clinics in the community-based study. *S*. *haematobium* was diagnosed by the urine filtration technique and *S*. *mansoni* was diagnosed by the Kato-Katz and formal ether concentration techniques. To evaluate the diagnostic performance of peptides to detect *S*. *mansoni* and *S*. *haematobium* IgG and IgM antibodies, sera from 135 participants (130 pre-school aged and primary school aged children aged 1–12 years and 5 participants older than 16) with a median age of 9 (IQR: 4–12) were purposively selected. Among the 135 participants 63% (n = 85) were females, 37% (n = 50) were males, 93% (n = 125) were from Mashonaland central a high schistosomiasis endemic area [2] and 7% (n = 10) which were designated the control group were from Harare a low schistosomiasis endemic area [2]. Participants from Mashonaland central (located 31°40′0” E longitude and 17°10′0” S latitude Northeast of Zimbabwe) were permanent residents of Mount Darwin and Shamva rural districts. Participants from Harare the capital city of Zimbabwe designated the control group were permanent residents of Glaudina a high-density urban area and they had no known history of *Schistosoma* infection or exposure to infested water sources.

### 2.3. Blood collection

Experienced local nurses collected approximately 5 ml of blood from each participant. The 5ml blood limit was within the guidelines for children issued by MRCZ research ethics committee. Blood samples were collected into red top serum tubes, stored overnight at 4°C and centrifuged for 10 minutes at 1000X to obtain sera that was used for the peptide microarray serological assays.

### 2.4. Parasitology examination

Urine and stool specimens were collected between 10: 00 am and 14:00 pm for optimal egg passage necessary for diagnosis of schistosomiasis. The specimens were stored away from direct sunlight until processing. The urine filtration technique was performed using 10 ml of urine to detect *S*. *haematobium* egg. The technique was repeated for three consecutive days to minimise misdiagnosis due to day-to-day egg variation. Infection intensity for *S*. *haematobium* was calculated as the arithmetic mean of the number of eggs per 10 ml of urine across all available samples. Participants were then classified into three infection intensity categories: negative (0 eggs per 10 ml urine), light (1–49 eggs per 10 ml of urine), and heavy (≥50 eggs per 10 ml of urine) [25].

The Kato-Katz technique was used to detect *S*. *mansoni* eggs from one stool sample using one standard 41.7 mg template for each participant. To determine the infection intensity the number of eggs were multiplied by a factor of 24 to scale the measurement to eggs per 1 g (EPG) of stool. Participants were grouped into four *S*. *mansoni* infection intensity categories: negative (0 EPG), light (1–99 EPG), moderate (100–399 EPG), and heavy (≥400 EPG) [25]. Additionally, *S*. *mansoni* was diagnosed using the formal ether concentration technique to improve accuracy. Participants were classified as infected if at least one parasitic egg was detected by either technique for both *S*. *haematobium* and *S*. *mansoni*.

### 2.5. Identification and selection of linear-cell epitopes

Two methods were used for the identification and selection of linear B-cell epitopes (peptides). These were a comprehensive literature search for published synthetic peptides and *in silico* prediction of novel peptides. For novel peptides, a literature search was conducted to identify recombinant proteins with good diagnostic candidate properties (excellent to outstanding diagnostic performances), ability to detect early and single worm infections, single-sex and non-fecund worm infections.

#### 2.5.1. Literature search

Five data bases PubMed, EMBASE, PsycInfo, CINAHL and the Cochrane library were systematically searched to identify *S*. *haematobium* and *S*. *mansoni* published peptides and recombinant proteins as previous described [26]. The databases were searched using variations and combinations of the following keywords: recombinant proteins, peptides, and schistosomiasis. The literature search was conducted from January 2000 to February 2022 without any language restrictions. The search strategy and results are shown in **S1 File**. Additionally, preprint databases MedRxiv and BioRxiv, websites of the WHO, the Schistosomiasis Control Initiative (SCI), and the Schistosomiasis Consortium for Operational Research and Evaluation (SCORE) were also searched.

#### *2*.*5*.*2*. *In silico* prediction and selection of novel peptides

The sequences and the alpha fold predicted structures of the identified recombinant proteins were obtained from Uniprot (https://www.uniprot.org/). SignalP 6.0 (https://services.healthtech.dtu.dk/service.php?SignalP-6.0) was used to identify the presence of signal peptides. Transmembrane domains were predicted using SOSUI (https://harrier.nagahama-i-bio.ac.jp/sosui/) and cellular localisation was predicted using WoLF Psort II (https://wolfpsort.hgc.jp/). DeepView/Swiss-PDB Viewer (www.expasy.org/spdbv/) was used for spatial location of the candidate peptides on the recombinant protein crystal structures.

Linear B cell epitopes were predicted using three different programs namely ABCpred (http://www.imtech.res.in/raghava/abcpred/), Bcepred (http://crdd.osdd.net/raghava/bcepred) and BepiPred 2.0 (www.cbs.dtu.dk/services/BepiPred/). ABCpred uses an artificial neural network, Bcepred predicts using physico-chemical properties of amino acids and BepiPred 2.0 uses a random forest algorithm. Peptides with the lowest sequence identity with human protein and proteins from other human pathogens were then selected using the NCBI Protein BLAST (https://blast.ncbi.nlm.nih.gov/Blast.cgi) to minimize potential cross reactivity. Peptides that had the highest probability of being B-cell epitopes and the lowest sequence identity with proteins from human and other pathogens were selected for inclusion on the peptide microarrays.

### 2.6. Peptide microarray content

The peptide microarrays were fabricated by PEPperPRINT GmbH (Heidelberg, Germany) (https://www.pepperprint.com/). The peptide microarrays contained 16 identical sub-arrays (copies). Each sub-array contained 122 *S*. *haematobium* and *S*. *mansoni* 9–16 amino acids long peptides printed randomly in duplicate. The peptides on each subarray were framed by HA (YPYDVPDYAG, 5 spots) and polio (YPYDVPDYAG, 3 spots) control peptides. Additionally, each sub-array was framed by glycine spacers (G spots).

### 2.7. Peptide microarrays immunoassay

Immunoassays were performed by PEPperPRINT GmbH (Heidelberg, Germany) as previously described [27,28]. Briefly, the immunoassays involved two steps on the same microarray. The first step was incubation with secondary antibody to identify false positive signals. The second step was incubation with serum and the secondary antibodies.

### 2.8. Identifying schistosome-specific antigenic peptides

The negative cut-off was determined by averaging the negative control readings (10 sera obtained from schistosomiasis unexposed and uninfected individuals with no prior history of *Schistosoma* infections) and adding 3 standard deviations. A positive response was defined as fluorescence intensity above the negative cut-off for each specific peptide for both IgG and IgM. Peptides for which at least one infected individual was positive were selected for further analysis. Statistical comparison between groups was done by the Kruskal-Wallis equality-of-populations rank test and *p-values* less 0.05 were considered statistically significant. Diagnostic accuracy was evaluated by receiver operating characteristic (ROC) curve analysis. The area under the ROC curve (AUC) was calculated to assess the overall diagnostic performance of peptides that were able to distinguish schistosome positives from schistosome negatives or healthy controls. Data curation and analyses were performed with Microsoft excel and Stata v17 (Stata, College Station, Texas, USA) respectively.

## 3 Results

### 3.1. Parasitology

Sera from ten participants from Harare confirmed to be egg negative for both *Schistosoma* infections by the urine filtration, Kato-Katz and formal ether techniques and with no known history of Schistosome infections or contact with contaminated water were considered the negative control. To evaluate the diagnostic performance of peptides to detect *S*. *haematobium* patient serum IgG and IgM antibodies, 43 (40 light infections and 3 heavy infections) serum samples collected from *S*. *haematobium* egg positive and 36 serum samples collected form *S*. *haematobium* egg negative patients were considered (**S2 File**). To evaluate the diagnostic performance of peptides to detect *S*. *mansoni* patient serum IgG and IgM antibodies, 46 and 37 serum samples collected from S. mansoni egg positive and negative patients respectively were considered. Among the serum samples collected positive patients 34 (15 light infections, 12 moderate infections and 7 heavy infections) were diagnosed by the Kato-Katz methods and 12 by the formal ether concentration technique (S2 File).

### 3.2. Characterisation of the native forms for *schistosome* recombinant proteins selected for novel peptide prediction

The literature search conducted to retrieve articles published between January 2000 and February 2022 identified a total of 18 *S*. *haematobium* and 10 *S*. *mansoni* recombinant proteins (S3 File). Due to limited funding only four were selected for the prediction of novel peptides, two for each Schistosome species. **Table 1** summarizes the characteristics of the proteins selected. MS3_10385 a serine protease inhibitor (SERPIN) and MS3_10186 a tetraspanin were selected for *S*. *haematobium* because they had excellent diagnostic performance across three platforms (urine IgG protein microarray, serum IgG ELISA and serum IgG protein microarray) with AUC values ranging from 0.86 to 0.93 (see **S3 File**). The native forms for the two recombinant proteins were predicted to have a signal peptide attached to them. Signal peptides are short N-terminal amino acid sequences that target proteins to the secretory pathways indicating that native forms of MS3_10385 and MS3_10186 are secretory proteins [29]. Native forms for both MS3_10385 and MS3_10186 were predicted to be soluble proteins indicating that they are not part of the transmembrane helix. Finally, WOLF Psort predicted the native forms of MS3_10385 and MS3_10186 to be extracellular or secreted proteins which are most likely to be involved in immunological.

**Table 1:** Properties and characteristics *S*. *haematobium* and *S*. *mansoni* recombinant proteins.

Description	Protein family	Gene name	Accession number	Source organism	Signal peptide	Transmembrane protein	Cellular localisation
Neuroserpin	SERPIN	MS3_10385	A0A095A7Z5	*S*. *haematobium*	Present	No (soluble protein)	Extracellular/ secreted
IPSE	Tetraspanin	MS3_10186	A0A095A2X3	*S*. *haematobium*	Present	No (soluble protein)	Extracellular/ secreted
RP26	SAPLIP	AAB81008	Q26536	*S*. *mansoni*	Present	Yes	Extracellular
SmSPI	SERPIN	SmSPI	G4LYU6	*S*. *mansoni*	Absent	No (soluble protein)	Mitochondria

Saposin-like proteins (SAPLIP)

Serine protease inhibitor (SERPIN)

For *S*. *mansoni* one protein SmSPI a SERPIN which had a sensitivity of 91.7% and a specificity of 93.3% on a serum IgG protein microarray was selected based on its diagnostic performance (**S3 File**). SmSPI was also selected because SAPLIPs have the potential to elicit a strong immune response as they may be continuously released into the host circulatory system in schistosome worm vomitus [30,31]. The second protein AAB81008 a saposin-like protein (SAPLIP) was selected due to its ability to detect early schistosome infections [32] and detect single worm infections which are missed by the egg detection methods. The native form of AAB81008 was predicted to have a signal peptide attached to it and the form of SmSPI was predicted not to possess a signal peptide. The native forms of AAB81008 and SmSPI were predicted to be membrane and soluble proteins respectively. Lastly, the native form of AAB81008 was predicted to be an extracellular protein and that of SmSPI was predicted to be located in the mitochondria.

### 3.3. Linear B-cell epitopes/peptides

Out of the 122 peptides *S*. *haematobium* and *S*. *mansoni* peptides selected for the peptide microarray immunoassays; 40.98% (n = 50), 22.95% (n = 28) and 21.31% (n = 26) were predicted with ABCpred, Bepi Pred 2 and Bcepred respectively and the remaining 14.75% (n = 18) obtained from literature (**S2 File**). The recombinant proteins AAB81008, SmSPI, 10385 and MS3_10186 were used to predict novel peptides. Tables 2 and 3, show that from the 122 peptides, only 14.75% (n = 18) were able to distinguish between schistosome positives, schistosome negatives and healthy controls. However, according to ROC curve analysis some peptides were inaccurate with AUC values less than 0.50.

**Table 2:** Diagnostic performance of peptides to detect *S*. *mansoni* patient serum IgG and IgM antibodies.

Peptide name	Linear sequence	Source organism	Antibody	Reference/control serum	p-value	ROC AUC	Std.error	95% Confidence interval		Peptide selection method
								Lower limit	Upper limit	
MS3_10186-119-35	VQCISESKRRRKYCRY	*S*. *haematobium*	IgG	*S*. *mansoni* negative	0.0328	0.6516	0.0607	0.53259	0.77058	ABCpred
				Healthy controls		0.6772	0.0796	0.52116	0.83319	
MS3_10186-123-131	SESKRRRKY	*S*. *haematobium*	IgG	*S*. *mansoni* negative	0.011	0.6219	0.0612	0.50202	0.74182	Bepi Pred 2
				Healthy controls		0.7690	0.0656	0.64093	0.89820	
MS3_10385-278-291	SIGVVDLFDPVKSD	*S*. *haematobium*	IgG	*S*. *mansoni* negative	0.0274	0.6475	0.0612	0.52755	0.76740	Bcepred
				Healthy controls		0.6924	0.0989	0.49858	0.88620	
MS3_10385-339-354	VDFHVTHPFICFIYDQ	*S*. *haematobium*	IgG	*S*. *mansoni* negative	0.0207	0.6301	0.0613	0.51002	0.75027	Bcepred
				Healthy controls		0.7261	0.0917	0.54641	0.90576	
SmSPI-177-193	MDDIPDDTGMILVNVF	*S*. *mansoni*	IgG	*S*. *mansoni* negative	0.0113	0.6539	0.0610	0.5344	0.77241	ABCpred
				Healthy controls		0.7239	0.0852	0.55700	0.89083	
SmSPI-378-388	NHPFICFIYDQ	*S*. *mansoni*	IgG	*S*. *mansoni* negative	0.01	0.6392	0.0612	0.51923	0.75926	Bcepred
				Healthy controls		0.7663	0.0834	0.602	0.92967	
SmSPI-362-378	IFVPISAVLPDIDFNV	*S*. *mansoni*	IgG	*S*. *mansoni* negative	0.0287	0.6486	0.0607	0.52965	0.76764	ABCpred
				Healthy controls		0.6957	0.0975	0.50460	0.88670	
AAB81008-19-30	INQPELEFGYKD	*S*. *mansoni*	IgG	*S*. *mansoni* negative	0.0019	0.6531	0.0599	0.53559	0.77052	Bepi Pred 2
				Healthy controls		0.8043	0.0660	0.67495	0.93374	
SmSP1-310-323	LKSMGIVDLFNPVA	*S*. *mansoni*	IgG	*S*. *mansoni* negative	0.0149	0.6525	0.0565	0.54170	0.77052	Bepi Pred 2
				Healthy controls		0.4402	0.0872	0.262929	0.61114	
SmSP1_359_372	TSPIFVPISAVLPD	*S*. *mansoni*	IgG	*S*. *mansoni* negative	0.004	0.7124	0.0560	0.60261	0.82218	Bepi Pred 2
				Healthy controls		0.5565	0.1083	0.34418	0.76887	
Smp 126160-438-452	LVTPESKYYSSLPGN	*S*. *mansoni*	IgG	*S*. *mansoni* negative	0.0022	0.7156	0.0559	0.605	0.82218	Literature
				Healthy controls		0.6761	0.0921	0.49562	0.85656	
MS3_10186-25-41	YCLRLYDGTYENGSYT	*S*. *haematobium*	IgG	*S*. *mansoni* negative	0.0035	0.7115	0.0580	0.59781	0.82522	ABCpred
				Healthy controls		0.6478	0.0970	0.4563	0.83802	
MS3_10186-40-49	PGSVCVPLIH	*S*. *haematobium*	IgG	*S*. *mansoni* negative	0.015	0.6604	0.0496	0.56315	0.75765	Bcepred
				Healthy controls		0.7098	0.0500	0.61173	0.80784	
AAB81008-181-196	STMDPDSACMTMHMCS	*S*. *mansoni*	IgG	*S*. *mansoni* negative	0.0226	0.6710	0.0593	0.55466	0.78729	ABCpred
				Healthy controls		0.5304	0.0987	0.33693	0.72394	
SmSPI-197-214	FWESPFEPHYTKIENFDI	*S*. *mansoni*	IgG	*S*. *mansoni* negative	0.05	0.5793	0.0643	0.45339	0.70525	Bepi Pred 2
				Healthy controls		0.7326	0.0775	0.58073	0.88448	
Smp 150390.1-216-230	SLPSNAHNNDNNSSD	*S*. *mansoni*	IgG	*S*. *mansoni* negative	0.0131	0.6713	0.0608	0.55217	0.79037	Literature
				Healthy controls		0.6989	0.0874	0.52761	0.87022	
MS3_10186-105-121	ISS1QKCVYGENGMVQ	*S*. *haematobium*	IgM	*S*. *mansoni* negative	0.0123	0.6898	0.0593	0.57352	0.80604	ABCpred
				Healthy controls		0.602	0.1067	0.46754	0.7501	
MS3_10186-40-49	PGSVCVPLIH	*S*. *haematobium*	IgM	*S*. *mansoni* negative	0.0158	0.6387	0.0582	0.52379	0.75353	Bcepred
				Healthy controls		0.7413	0.0644	0.61628	0.85607	

The peptide name was derived from the gene name followed by the position of the peptide amino acid sequence on the protein.

p-values were determined by the Kruskal-Wallis equality-of-populations rank test and p-values less than 0.05 were considered significant.

**Table 3:** Diagnostic performance of peptides to detect *S*. *haematobium* patient serum IgG and IgM antibodies.

Peptide name	Linear sequence	Source organism	Antibody	Reference/control serum	*p-value*	ROC AUC	Std.error	Confidence interval		Peptide selection method
								Lower limit	Upper limit	
SmSPI-177-193	MDDIPDDTGMILVNVF	*S*. *mansoni*	IgG	*S*. *haematobium* negative	0.032	0.372	0.0647	0.24602	0.49946	ABCpred
				Healthy controls		0.6302	0.1065	0.42153	0.83894	
AAB81008-19-30	INQPELEFGYKD	*S*. *mansoni*	IgG	*S*. *haematobium* negative	0.0384	0.4464	0.0666	0.31587	0.57690	Bepi Pred 2
				Healthy controls		0.7326	0.0810	0.5786	0.8916	
SmSP1-165-181	DQQSNGLLEKFFMDDIP	*S*. *mansoni*	IgG	*S*. *haematobium* negative	0.0373	0.4092	0.0650	0.28180	0.53668	Bepi Pred 2
				Healthy controls		0.6674	0.1017	0.46811	0.86678	
AAB81008-19-30	INQPELEFGYKD	*S*. *mansoni*	IgM	*S*. *haematobium* negative	0.0294	0.3692	0.0640	0.24367	0.49470	Bepi Pred 2
				Healthy controls		0.6116	0.1053	0.40522	0.81804	

The peptide name was derived from the gene name followed by the position of the peptide amino acid sequence on the protein.

p-values were determined by the Kruskal-Wallis equality-of-populations rank test and p-values less than 0.05 were considered significant.

### 3.4. Diagnostic performance for discriminating *S*. *mansoni* positives from healthy controls

**Fig 1:**
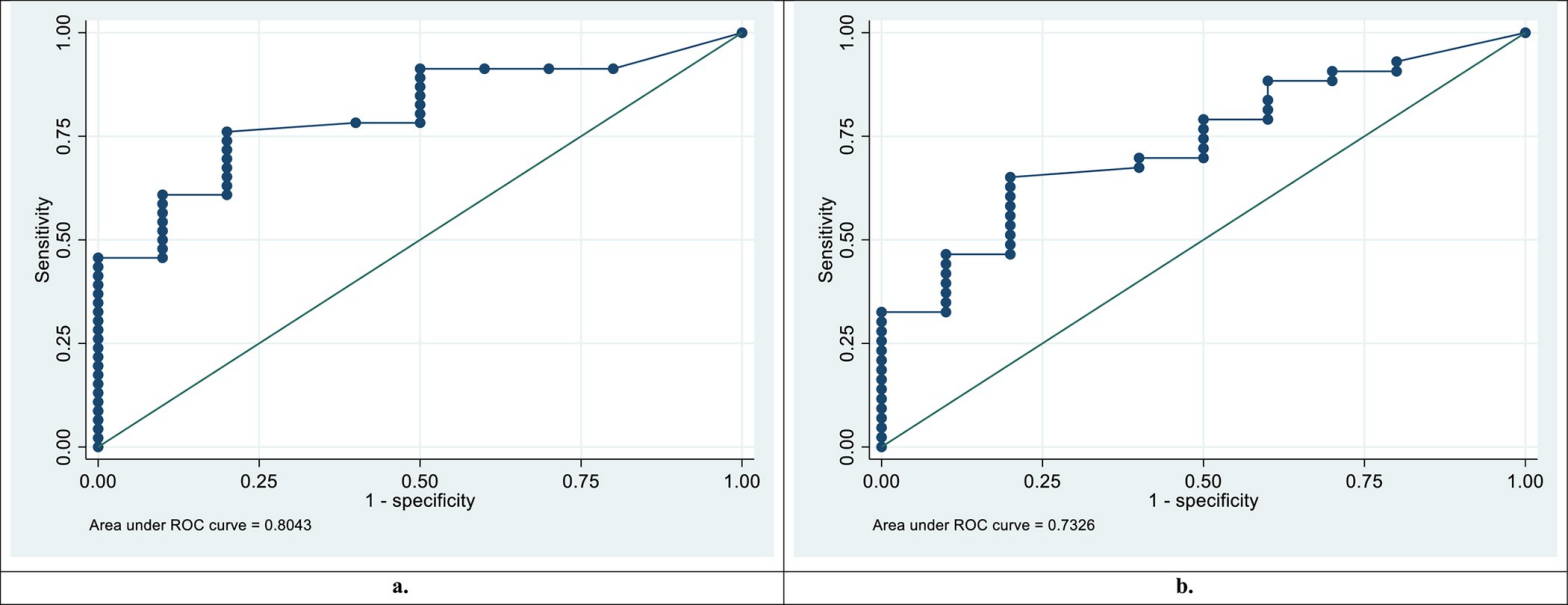
**Receiver Operating Characteristic (ROC) curves for Peptide AAB81008-19-30 a. Peptide AAB81008-19-30** ROC curve and area under the ROC curve (AUC) for discrimination of *S*. *mansoni* positives from healthy controls for IgG. b. **Peptide AAB81008-19-30** ROC curve and area under the ROC curve (AUC) for discrimination of *S*. *haematobium* positives from healthy controls for IgG.

**Fig 2:**
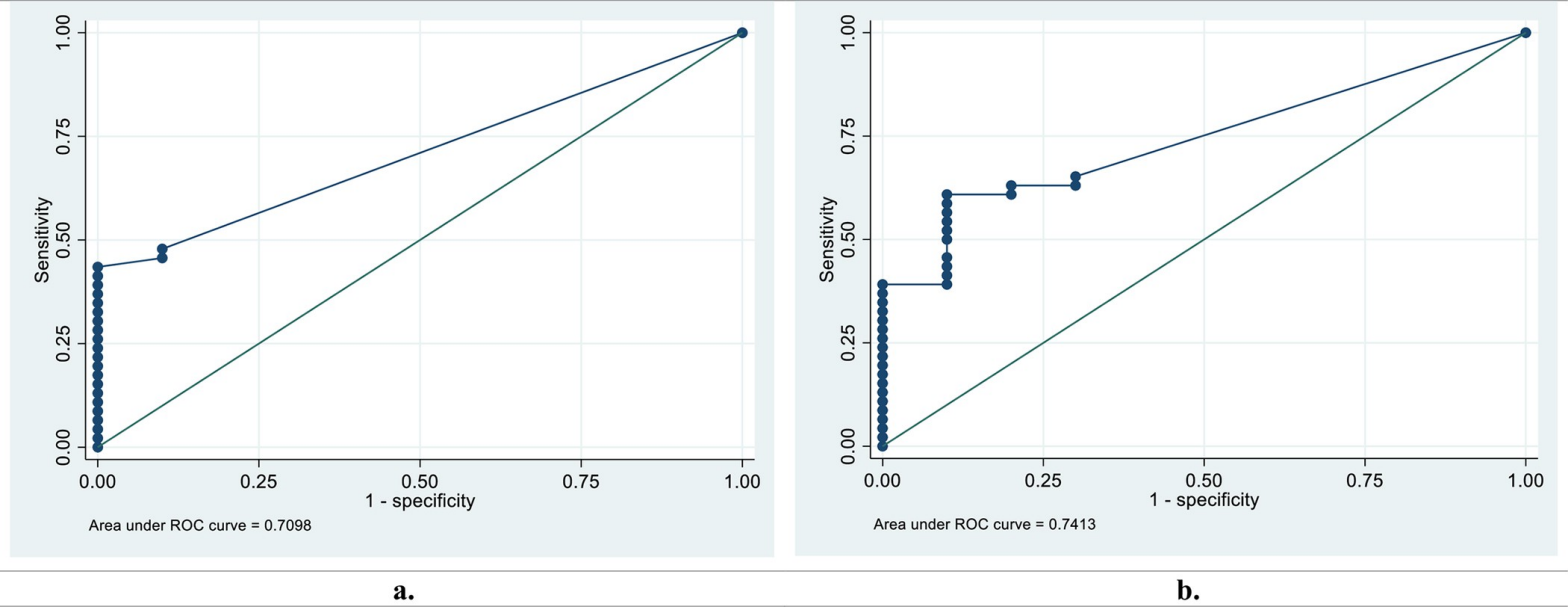
**Receiver Operating Characteristic (ROC) curves for Peptide MS3 10186-40-49 a. Peptide MS3_10186-40-49** ROC curve and area under the ROC curve (AUC) for discrimination of *S*. *mansoni* positives from healthy controls for IgG. b. **Peptide MS3_10186-40-49** ROC curve and area under the ROC curve (AUC) for discrimination of *S*. *haematobium* positives from healthy controls for IgM.

Out of the 16 peptides that were able to distinguish *S*. *mansoni* positive sera from schistosome negative or healthy controls sera, only peptide AA81008-19-30 had an excellent diagnostic performance for discriminating *S*. *mansoni* positives from healthy controls with an AUC value of 0.8043 for IgG peptide microarray (**Fig 1**). Six peptides MS3_10186-123-131, MS3_10385-339-354, SmSPI_177–193, SmSPI_379–388, MS3_10186-40-49 and SmS_197–214 had acceptable diagnostic performances for discriminating *S*. *mansoni* positives from healthy controls with AUC values ranging from 0.7098 to 0.7763 for IgG peptide microarray (**S3 File**). For IgM peptide microarray the ROC curve analysis for discriminating *S*. *mansoni* positives from the healthy controls yielded AUC values of 0.602 and 0.7413 for MS3 10186_105–121 and MS3 10186_40–49 respectively (**S3 File**). Peptide MS3_10186-40-49 had an acceptable diagnostic performance for discriminating *S*. *mansoni* positives from healthy controls for both IgG and IgM (**Fig 2**).

### 3.5. Diagnostic performance for discriminating *S*. *mansoni* positives from *S*. *mansoni* negatives

**Fig 3:**
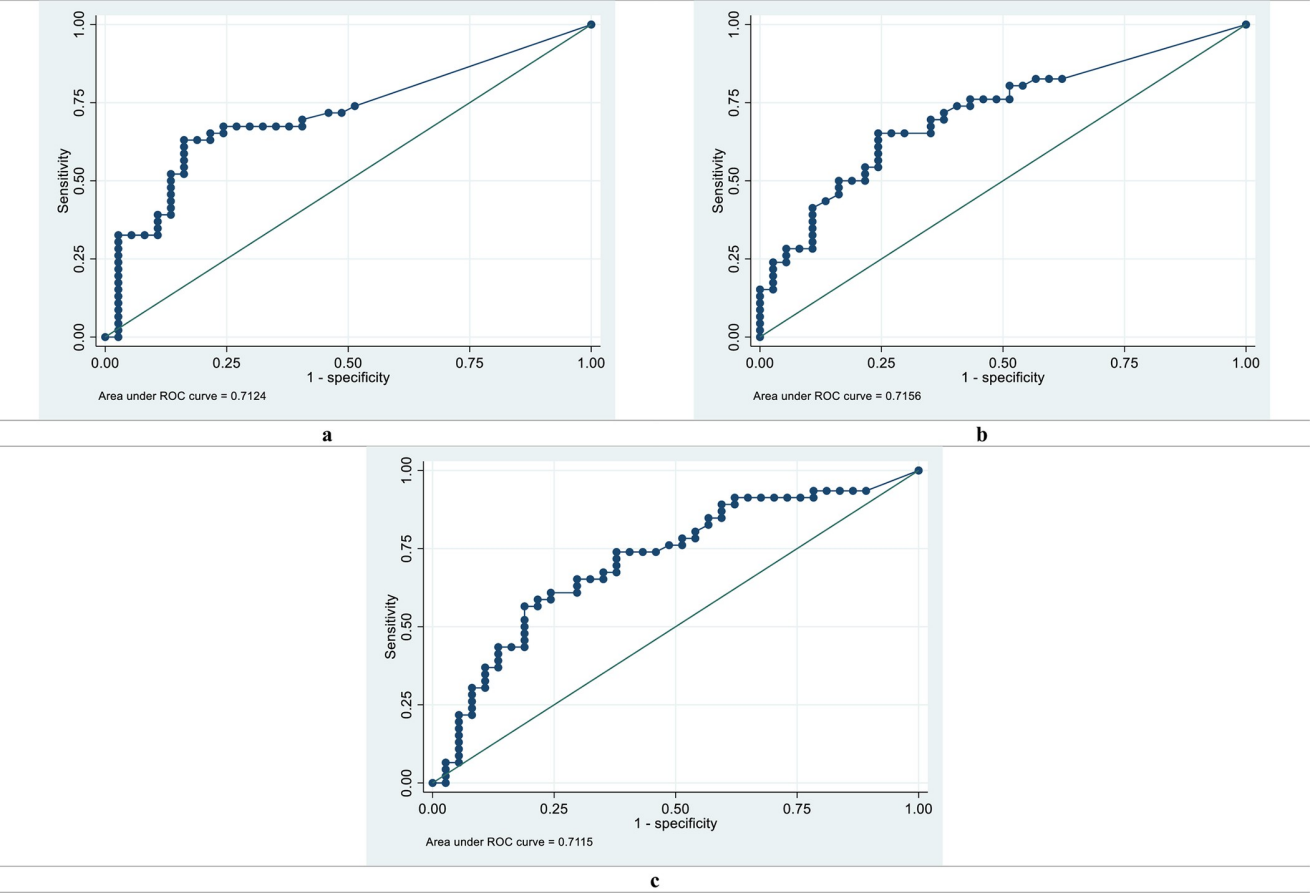
Receiver operating characteristic (ROC) curves and area under the ROC curves (AUC) for discriminating *S*. *mansoni* positives from *S*. *mansoni* negatives for IgG peptide microarray. a. **Peptide SmpSPI-359-372** b. **Peptide Smp 126160-438-452** c. **Peptide MS3_10186-25-41**.

Three peptides SmSPI-359-372, Smp 126160-438-452 and MS3_10186-25-41 had acceptable diagnostic performances for discriminating *S*. *mansoni* positives from *S*. *mansoni* negatives with AUC values of 0.7124, 0.7156, 0.7115 respectively for IgG peptide microarray (**Fig 3**). For IgM peptide microarray the ROC curve analysis for discriminating the *S*. *mansoni* positives from the *S*. *mansoni* negatives yielded AUC values of 0.6898 and 0.6387 for MS3_10186-105-121 and MS3_10186-40-49 respectively (**S5 and S6 Files**).

### 3.6. Diagnostic performance for discriminating *S*. *haematobium* positives from *S*. *haematobium* negatives and healthy controls

Peptides SmSPI-177-193, AAB81008-19-30, SmSP1-165-181 and AAB81008-19-30 had inaccurate diagnostic performances for discriminating *S*. *haematobium* positives from *S*. *haematobium* negatives for both IgG and IgM peptide microarray with AUC values under 0. 5 (**S5 and 6Files**). Peptides SmSPI-177-193 and SmSPI-165-181 had poor diagnostic performances for discriminating *S*. *haematobium* positives from healthy controls for IgG peptide microarray. Peptide AAB81008-19-30 had an acceptable diagnostic performance for discriminating *S*. *haematobium* positives from healthy controls with an AUC value of 0.7326 for IgG peptide microarray. Peptide AAB81008-19-30 was able to discriminate both *S*. *haematobium* and *S*. *mansoni* positives from healthy controls (**Fig 1**).

### 3.7. Spatial location of novel peptides with good and acceptable diagnostic performance on the recombinant proteins 3D structures

**Fig 4:**
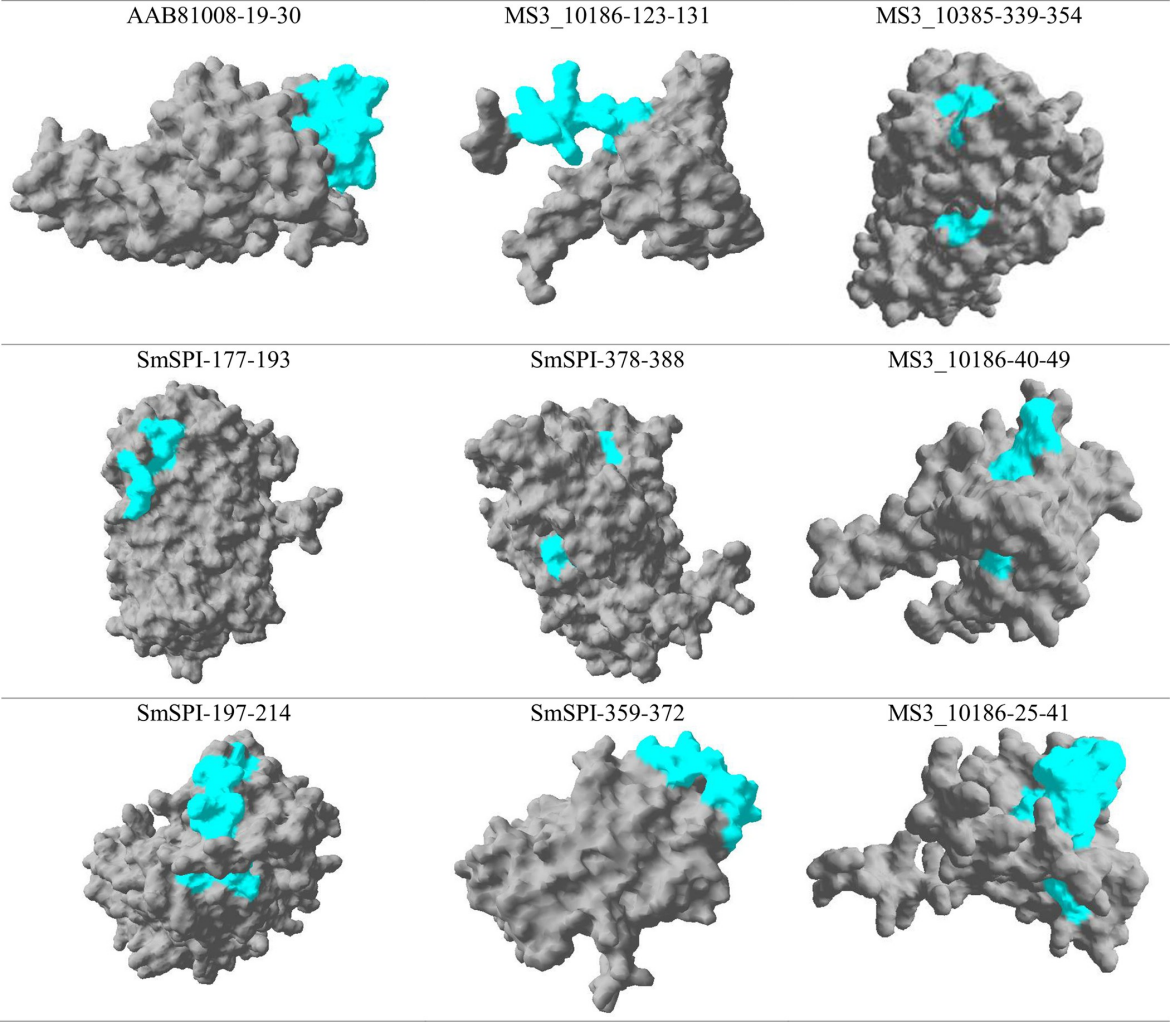
Spatial location of novel peptides with good and acceptable diagnostic performances on the recombinant proteins 3D structures. DeepView/Swiss-PDB Viewer was used to determine the spatial location of the peptides on the crystal structure of recombinant proteins (MS3_10385; MS3_10186; AAB81008-19-30 and SmSPI).

Understanding the spatial location of peptides within a protein crystal structure is crucial for unravelling the intricacies of molecular interactions and biological functions. In our study DeepView/Swiss-PDB Viewer (www.expasy.org/spdbv/) was used for spatial location of the candidate peptides on the protein crystal structures of MS3_10385; MS3_10186; AAB81008 and SmSPI (**Fig 4**). Peptides AAB1008-19-30, MS3_10186-123-131 and MS3_10385-359-372 were located at the exterior surface of their respective protein structures. Most of the target peptide amino acid sequence was located at the exterior surface of the protein with a few amino acid residues encapsulated within the protein for peptides SmSPI_197–214, MS3_10186-40-49 and MS3_10186-25-41. Lastly, most of the amino acid residues for peptides for SmSPI_177–193, SmSPI_378–388 and MS3_10385-339-354 were encapsulated within the protein structure and only a few amino acid residues were at the exterior surface of the protein.

## 4. Discussion

As countries target the elimination of schistosomiasis as a public health problem and as the prevalence of the disease decreases due to MDA campaigns, it has become apparently clear that more sensitive field-applicable diagnostics are needed for the effective management and surveillance of schistosome infections as advocated by the WHO [5,10,12]. This is particularly important in low endemicity areas, where microscope-based diagnostic methods may underestimate the true prevalence of the disease [11,33]. This background has provided an impetus for this study in the identification of peptides that can be employed in the development of antibody-based diagnostic tools using an immunoinformatic approach and peptide microarray immunoassay validation.

Ten peptides (one with an excellent diagnostic performance and nine with acceptable diagnostic performances) were identified using the approach. These findings validate studies by Carvalho and colleagues, 2022 and Lopes and colleagues, 2017 who previously used immunoinformatic approaches and immuno-assay validation to identify *S*. *haematobium* and *S*. *mansoni* peptides with diagnostic potential [18,19]. Eighteen previously published peptides were included on the peptide microarray, 10 from our previous study Vengesai and colleagues, 2022 [27] and 8 peptides from two studies by Carvalho and Colleagues, 2022 and Lopes and Colleagues, 2017 [18,19]. Despite the two studies showing that peptides Sm168240, Smp_136560 (1564–1578), Smp_126160(438–452), Sm140560, Sm041370, Smp_180240(339–353), Smp_150390.1(216–230), Smp_093840(219–233) had AUC values ranging from 0.76 (95% CI 0.6024–0.9213) to 0.99 (95% CI 0.987–100) results from the current study show that only one published peptide Smp 126160-438-452 had an acceptable diagnostic performance with an AUC value of 0.7115 (95% CI 0.605–0.82218). These findings may be attributed to the fact that peptide microarray immunoassays were used in the determination of antibody reactivity against the peptides whilst in the other two studies [18,19] antibody reactivity was investigated using peptide based serum IgG ELISA. Pertaining to the other 10 published peptides XP_035587815.1-269-283, XP_012797374.1-78-92, AAZ29530.1-25-29, XP_012799745.1-16-30, P20287.1-58-72, AAA29903.1-222-237, P09841.3-6-20, AAA29900.1-145-159, P09792.1-29-43, XP_035588858.1-206-220 none of the peptides showed a clear discrimination between the schistosome infected and uninfected groups and these results are in agreement with findings from our previous study [27].

The peptide microarray immunoassay identified one peptide AAB81008-19-30 which showed cross reactivity with *S*. *haematobium* and *S*. *mansoni* IgG. The cross-reactivity of peptide AAB81008-19-30 may have important consequences for species-specific and species transcending immunity [34]. Furthermore, Peptide AAB81008-19-30 was derived from RP26 a SAPLIP which is expressed at the cercarial stages [32] thereby, making it a good vaccine candidate to fight both *S*. *haematobium* and *S*. *mansoni* early infections.

Despite, the egg detection methods being able to detect active infections and provide quantitative information on infection intensity and worm burden, they are not able to detect early, single, single-sex and non-fecund schistosome worm infections [35]. Undetected, and untreated cases pose as drivers for persistent hotspots in the case of early/recent infections, threatening the successful elimination of schistosomiasis by serving as infection reservoirs and re-infecting their communities [9,36,37]. The single-sex and non-fecund infections can be drivers for persistent morbidity. RP26 is also expressed at schistosomula and immature worms’ stages, and not in the egg stage [32] suggesting that peptide AA81008-19-30 has the ability to detect early infections and exposure to single worm, single-sex and non-fecund worm infections. Furthermore, peptide-based antibody/serological assays have been shown to be capable of simultaneous multiplex diagnosis of different pathogens with a single patient serum sample [38–40]. The possibility of testing the same serum sample simultaneously for the presence of antibodies against both *S*. *haematobium* and *S*. *mansoni* is an added value particularly in seroprevalence studies linked to control programs in endemic areas where these two parasites coexist.

### 4.1. Limitations and recommendations

Ideally, to properly evaluate the performance of the identified peptides their diagnostic accuracy needs to be compared to a reference standard test. The reference standard test should be able to discriminate the true positives and true negatives. The absence of a standard reference test or absolute knowledge of the true positives was a significant limitation in the present study [11,41–43]. Despite the inappropriateness of doing so, Kato-Katz and urine filtration techniques were used as the reference tests in the current study. The techniques lack sensitivity especially in low endemicity settings; hence it is expected that some positive individuals were misdiagnosed as false-negative [10,11]. This might have contributed to the low diagnostic performances of some identified peptides. To circumvent this limitation, we propose use of the latent class analysis previously described by Mesquita and colleagues, 2022, which combines multiple direct and indirect tests to construct a standard reference outcome [11]. However, if the LCA is based on the same principle, for example antibody detection assays, and only a weak contribution of parasitological and/or molecular exams, the results might be misleading. Therefore, we also propose inclusion of a composite reference standard based on the direct tests [43].

The majority of the participants had light *S*. *haematobium infections* and light to moderate *S*. *mansoni* infections. The peptides generally had good specificities and poor sensitives against these light to moderate infections probably due to low antibody levels, ultimately making the peptides poor diagnostic candidates. However, the possibility of the construction of multi-epitope chimeric proteins could improve the sensitivity of these peptides [19,44]. A study by Oliveira and colleagues used a multi-epitope chimeric protein constructed from a pool of 5 epitopes/peptides derived from *S*. *mansoni* proteins in a serum-based IgG ELISA and demonstrated high diagnostic performances compared to the single peptides [17].

Unfortunately, like most currently available serological tests the peptides identified are not useful for monitoring and evaluation programmes because antibodies to schistosome infections remain detectable long after treatment [45]. This limits the clinical value of antibody detection for confirmation of the success of chemotherapy, since specific antibodies continue to be present long after the worms have disappeared [14,46]. There may be certain antigens (for example peptides) to which certain antibody isotype subclasses like IgG_4_, disappear more rapidly [45]. These antibodies can be targeted to circumvent the limitation associated with persisting antibodies. According to the WHO, serological and immunological tests are useful for showing exposure to schistosome infection in people living in non-endemic or low-transmission [47]. Alternatively, the identified peptides can be used to develop serological tools for showing exposure to infection for people living in non-endemic and low-transmission areas.

As urogenital and intestinal schistosomiasis are poverty related diseases prevalent in resource-limited settings, cost is a major factor for developing diagnostic tools for the diseases [48]. Therefore, we recommend that peptides discovered by peptide microarray technologies be transferred to point-of-care lateral flow platforms. Lastly, as previously described potential peptides should be validated with extremely well characterized samples with multiple infections, as well as true negative controls and a good positive reference (characterised with parasitological, molecular and antigen-based assays) [43,49].

### 4.2. Conclusion

In conclusion, one peptide with a good diagnostic performance and nine peptides with acceptable diagnostic performance were identified using the immunoinformatic approach and peptide microarray validation. Identified peptides maybe be used to develop diagnostic tools for showing exposure to schistosome infection in people living in non-endemic or low-transmission areas. Peptide AA81008-19-30 may be used to develop a tool for diagnosis of early *S*. *mansoni* worm infections. However, there is need for validation of the findings with true negative controls from a non-endemic country and a good reference tool.

## Supporting information

S1 File Literature search strategies and results for the identification of *S*. *haematobium* and S. mansoni peptides and recombinant proteins published between 2000 and 2022. (PDF)

S2 File *S*. *haematobium* and *S*. *mansoni* demographics, parasitology and peptide microarray immunoassay data sets. (XLSX)

S3 File Diagnostic performance of recombinant proteins to detect *S*. *haematobium* and *S*. *mansoni* patient IgG and IgM antibodies. (PDF)

S4 File *S*. *haematobium* and *S*. *mansoni* linear B-cell epitopes. (XLSX)

S5 File Receiver operating characteristics (ROC) curve and area under the ROC curve (AUC) to detect *S*. *mansoni* (**a-q**) and *S*. *haematobium* (**r-t**) patient serum IgG.
(PDF)

S6 File Receiver operating characteristics (ROC) curve and area under the ROC curve (AUC) to detect *S*. *mansoni* (**a-b**) and *S*. *haematobium* (**c**) patient serum IgM.
(PDF)

## References

[1] Schistosomiasis [Internet]. [cited 2023 May 24]. Available from: https://www.who.int/news-room/fact-sheets/detail/schistosomiasis.

[2] Midzi N, Mduluza T, Chimbari MJ, Tshuma C, Charimari L, Mhlanga G, et al. Distribution of Schistosomiasis and Soil Transmitted Helminthiasis in Zimbabwe: Towards a National Plan of Action for Control and Elimination. Kabatereine NB, editor. PLoS Negl Trop Dis [Internet]. 2014 Aug 14 [cited 2020 Mar 3];8(8):e3014. Available from: http://dx.plos.org/10.1371/journal.pntd.0003014. doi: 10.1371/journal.pntd.0003014 25121489 PMC4133179

[3] Nausch N, Dawson EM, Midzi N, Mduluza T, Mutapi F, Doenhoff MJ. Field evaluation of a new antibody-based diagnostic for Schistosoma haematobium and S. mansoni at the point-of-care in northeast Zimbabwe [Internet]. 2014 [cited 2020 May 20]. Available from: http://www.biomedcentral.com/1471-2334/14/165. 10.1186/1471-2334-14-165 PMC4021455 24666689

[4] Vengesai A, Naicker T, Kasambala M, Midzi H, Mduluza-Jokonya T, Rusakaniko S, et al. Clinical utility of peptide microarrays in the serodiagnosis of neglected tropical diseases in sub-Saharan Africa: protocol for a diagnostic test accuracy systematic review. 2021 Jul [cited 2022 Jan 9];11(7):e042279. Available from: https://pubmed.ncbi.nlm.nih.gov/34330850/. doi: 10.1136/bmjopen-2020-042279 34330850 PMC8327806

[5] Archer J, Barksby R, Pennance T, Rostron P, Bakar F, Knopp S, et al. Analytical and Clinical Assessment of a Portable, Isothermal Recombinase Polymerase Amplification (RPA) Assay for the Molecular Diagnosis of Urogenital Schistosomiasis. Mol 2020, Vol 25, Page 4175 [Internet]. 2020 Sep 11 [cited 2023 May 24];25(18):4175. Available from: https://www.mdpi.com/1420-3049/25/18/4175/htm. doi: 10.3390/molecules25184175 32933094 PMC7570534

[6] Lim MD, Brooker SJ, Belizario VY, Gay-Andrieu F, Gilleard J, Levecke B, et al. Diagnostic tools for soil-transmitted helminths control and elimination programs: A pathway for diagnostic product development. PLoS Negl Trop Dis [Internet]. 2018 Mar 1 [cited 2023 May 24];12(3):e0006213. Available from: https://journals.plos.org/plosntds/article?id=10.1371/journal.pntd.0006213. doi: 10.1371/journal.pntd.0006213 29494581 PMC5832200

[7] Mu Y, Gordon CA, Olveda RM, Ross AG, Olveda DU, Marsh JM, et al. Identification of a linear B-cell epitope on the Schistosoma japonicum saposin protein, SjSAP4: Potential as a component of a multi-epitope diagnostic assay. PLoS Negl Trop Dis [Internet]. 2022 [cited 2023 May 24];16(7):e0010619. Available from: https://journals.plos.org/plosntds/article?id=10.1371/journal.pntd.0010619. doi: 10.1371/journal.pntd.0010619 35816547 PMC9302751

[8] Hoermann J, Kuenzli E, Schaefer C, Paris DH, Bühler S, Odermatt P, et al. Performance of a rapid immuno-chromatographic test (Schistosoma ICT IgG-IgM) for detecting Schistosoma-specific antibodies in sera of endemic and non-endemic populations. PLoS Negl Trop Dis [Internet]. 2022 [cited 2023 May 24];16(5):e0010463. Available from: https://journals.plos.org/plosntds/article?id=10.1371/journal.pntd.0010463. 35622871 10.1371/journal.pntd.0010463 PMC9212132

[9] Menezes DL, Santos CT de J, Oliveira YLDC, Campos, Negrão-Corrêa DA, Geiger SM, et al. Accuracy Study of Kato-Katz and Helmintex Methods for Diagnosis of Schistosomiasis Mansoni in a Moderate Endemicity Area in Sergipe, Northeastern Brazil. Diagnostics 2023, Vol 13, Page 527 [Internet]. 2023 Jan 31 [cited 2023 May 24];13(3):527. Available from: https://www.mdpi.com/2075-4418/13/3/527/htm. 10.3390/diagnostics13030527 PMC9914664 36766631

[10] Colley DG, King CH, Kittur N, Ramzy RMR, Secor WE, Fredericks-James M, et al. Evaluation, Validation, and Recognition of the Point-of-Care Circulating Cathodic Antigen, Urine-Based Assay for Mapping Schistosoma mansoni Infections. Am J Trop Med Hyg [Internet]. 2020 May 12 [cited 2023 May 24];103(1_Suppl):42–9. Available from: https://www.ajtmh.org/view/journals/tpmd/103/1_Suppl/article-p42.xml. doi: 10.4269/ajtmh.19-0788 32400347 PMC7351311

[11] Mesquita SG, Caldeira RL, Favre TC, Massara CL, Beck LCNH, Simões TC, et al. Assessment of the accuracy of 11 different diagnostic tests for the detection of Schistosomiasis mansoni in individuals from a Brazilian area of low endemicity using latent class analysis. Front Microbiol. 2022 Dec 15;13:4683. doi: 10.3389/fmicb.2022.1048457 36590409 PMC9797737

[12] Pearson MS, Tedla BA, Mekonnen GG, Proietti C, Becker L, Nakajima R, et al. Immunomics-guided discovery of serum and urine antibodies for diagnosing urogenital schistosomiasis: a biomarker identification study. The Lancet Microbe [Internet]. 2021 Nov 1 [cited 2023 May 26];2(11):e617–26. Available from: http://www.thelancet.com/article/S2666524721001506/fulltext. doi: 10.1016/S2666-5247(21)00150-6 34977830 PMC8683377

[13] Imai N, Rujeni N, Nausch N, Bourke CD, Appleby LJ, Cowan G, et al. Exposure, infection, systemic cytokine levels and antibody responses in young children concurrently exposed to schistosomiasis and malaria. Parasitology. 2011 Oct;138(12):1519–33. doi: 10.1017/S0031182011001181 21813042 PMC3178872

[14] Ogongo P, Kariuki TM, Wilson RA. Diagnosis of schistosomiasis mansoni: an evaluation of existing methods and research towards single worm pair detection. Parasitology [Internet]. 2018 Sep 1 [cited 2022 Mar 12];145(11):1355–66. Available from: https://www.cambridge.org/core/journals/parasitology/article/abs/diagnosis-of-schistosomiasis-mansoni-an-evaluation-of-existing-methods-and-research-towards-single-worm-pair-detection/552001BF61F171FB60F11577DC42280A. doi: 10.1017/S0031182018000240 29506583

[15] Ma L, Zhao W, Hou X, Liu M, Li Y, Shen L, et al. Identification of linear epitopes in SjSP-13 of Schistosoma japonicum using a GST-peptide fusion protein microplate array. Parasites and Vectors [Internet]. 2019 Oct 30 [cited 2020 Nov 22];12(1). Available from: https://pubmed-ncbi-nlm-nih-gov.ukzn.idm.oclc.org/31666115/. doi: 10.1186/s13071-019-3767-2 31666115 PMC6822365

[16] Falconi-Agapito F, Kerkhof K, Merino X, Bakokimi D, Torres F, Van Esbroeck M, et al. Peptide Biomarkers for the Diagnosis of Dengue Infection. Front Immunol. 2022 Jan 26;13:52. 10.3389/fimmu.2022.793882 PMC8826428 35154111

[17] de Oliveira EJ, Kanamura HY, Takei K, Hirata RDC, Valli LCP, Nguyen NY, et al. Synthetic peptides as an antigenic base in an ELISA for laboratory diagnosis of schistosomiasis mansoni. Trans R Soc Trop Med Hyg [Internet]. 2008 Apr 1 [cited 2022 Feb 15];102(4):360–6. Available from: https://pubmed.ncbi.nlm.nih.gov/18314149/. doi: 10.1016/j.trstmh.2007.11.008 18314149

[18] Carvalho GBF, Resende DM, Siqueira LMV, Lopes MD, Lopes DO, Coelho PMZ, et al. Selecting targets for the diagnosis of Schistosoma mansoni infection: An integrative approach using multi-omic and immunoinformatics data. PLoS One [Internet]. 2017 Aug 1 [cited 2022 Jan 9];12(8):e0182299. Available from: https://journals.plos.org/plosone/article?id=10.1371/journal.pone.0182299. doi: 10.1371/journal.pone.0182299 28817585 PMC5560627

[19] Lopes MD, Oliveira FM, Coelho IEV, Passos MJF, Alves CC, Taranto AG, et al. Epitopes rationally selected through computational analyses induce T-cell proliferation in mice and are recognized by serum from individuals infected with Schistosoma mansoni. Biotechnol Prog [Internet]. 2017 May 1 [cited 2022 Feb 14];33(3):804–14. Available from: https://pubmed.ncbi.nlm.nih.gov/28371522/. doi: 10.1002/btpr.2463 28371522

[20] Van Regenmortel MHV. Structural and functional approaches to the study of protein antigenicity [Internet]. Vol. 10, Immunology Today. Immunol Today; 1989 [cited 2021 Jun 14]. p. 266–72. Available from: https://pubmed.ncbi.nlm.nih.gov/2478146/. doi: 10.1016/0167-5699(89)90140-0 2478146

[21] Giacò L, Amicosante M, Fraziano M, Gherardini PF, Ausiello G, Helmer-Citterich M, et al. B-Pred, a structure based B-cell epitopes prediction server. Adv Appl Bioinforma Chem [Internet]. 2012 Jul 25 [cited 2021 Jun 14];5(1):11–21. Available from: doi: 10.2147/AABC.S30620 22888263 PMC3413014

[22] Vengesai A, Kasambala M, Mutandadzi H, Mduluza-Jokonyaid TL, Mduluzaid T, Naicker T, et al. Scoping review of the applications of peptide microarrays on the fight against human infections. PLoS One [Internet]. 2022 Jan [cited 2022 Feb 13];17(1):e0248666. Available from: https://journals.plos.org/plosone/article?id=10.1371/journal.pone.0248666. doi: 10.1371/journal.pone.0248666 35077448 PMC8789108

[23] Sanchez-Lockhart M, Reyes DS, Gonzalez JC, Garcia KY, Villa EC, Pfeffer BP, et al. Qualitative Profiling of the Humoral Immune Response Elicited by rVSV-ΔG-EBOV-GP Using a Systems Serology Assay, Domain Programmable Arrays. Cell Rep [Internet]. 2018 Jul 24 [cited 2020 Nov 26];24(4):1050–1059.e5. Available from: 10.1016/j.celrep.2018.06.077. 30044972

[24] Sanchez-Trincado JL, Gomez-Perosanz M, Reche PA. Fundamentals and Methods for T- and B-Cell Epitope Prediction [Internet]. Vol. 2017, Journal of Immunology Research. Hindawi Limited; 2017 [cited 2021 Jan 14]. Available from: https://pubmed.ncbi.nlm.nih.gov/29445754/. 10.1155/2017/2680160 PMC5763123 29445754

[25] WHO GUIDELINE on control and elimination of human schistosomiasis. 35235279

[26] Vengesai A, Muleya V, Midzi H, Tinago TV, Chipako I, Manuwa M, et al. Diagnostic performances of Schistosoma haematobium and Schistosoma mansoni recombinant proteins, peptides and chimeric proteins antibody based tests. Systematic scoping review. PLoS One. 2023 Mar 1;18(3 March). doi: 10.1371/journal.pone.0282233 36862712 PMC9980832

[27] Vengesai A, Naicker T, Midzi H, Kasambala M, Mduluza-Jokonya TL, Rusakaniko S, et al. Multiplex peptide microarray profiling of antibody reactivity against neglected tropical diseases derived B-cell epitopes for serodiagnosis in Zimbabwe. PLoS One [Internet]. 2022 Jul 1 [cited 2023 Jan 31];17(7):e0271916. Available from: https://journals.plos.org/plosone/article?id=10.1371/journal.pone.0271916. doi: 10.1371/journal.pone.0271916 35867689 PMC9307155

[28] Vengesai A, Naicker T, Midzi H, Kasambala M, Muleya V, Chipako I, et al. Peptide microarray analysis of in-silico predicted B-cell epitopes in SARS-CoV-2 sero-positive healthcare workers in Bulawayo, Zimbabwe. Acta Trop [Internet]. 2023 Feb 1 [cited 2023 Jan 31];238:106781. Available from: https://linkinghub.elsevier.com/retrieve/pii/S0001706X22004727. doi: 10.1016/j.actatropica.2022.106781 36460093 PMC9705268

[29] Teufel F, Almagro Armenteros JJ, Johansen AR, Gíslason MH, Pihl SI, Tsirigos KD, et al. SignalP 6.0 predicts all five types of signal peptides using protein language models. Nat Biotechnol 2022 407 [Internet]. 2022 Jan 3 [cited 2024 Mar 25];40(7):1023–5. Available from: https://www.nature.com/articles/s41587-021-01156-3. doi: 10.1038/s41587-021-01156-3 34980915 PMC9287161

[30] Don TA, Bethony JM, Loukas A. Saposin-like proteins are expressed in the gastrodermis of Schistosoma mansoni and are immunogenic in natural infections. Int J Infect Dis [Internet]. 2008 Nov [cited 2022 Aug 18];12(6). Available from: https://pubmed.ncbi.nlm.nih.gov/18571965/. doi: 10.1016/j.ijid.2007.10.007 18571965

[31] Mu Y, Gordon CA, Olveda RM, Ross AG, Olveda DU, Marsh JM, et al. Identification of a linear B-cell epitope on the Schistosoma japonicum saposin protein, SjSAP4: Potential as a component of a multi-epitope diagnostic assay. Morassutti A, editor. PLoS Negl Trop Dis [Internet]. 2022 Jul 11 [cited 2022 Aug 18];16(7):e0010619. Available from: https://journals.plos.org/plosntds/article?id=10.1371/journal.pntd.0010619. doi: 10.1371/journal.pntd.0010619 35816547 PMC9302751

[32] Tanigawa C, Fujii Y, Miura M, Nzou SM, Mwangi AW, Nagi S, et al. Species-Specific Serological Detection for Schistosomiasis by Serine Protease Inhibitor (SERPIN) in Multiplex Assay. PLoS Negl Trop Dis [Internet]. 2015 [cited 2020 May 20];9(8):e0004021. Available from: https://journals.plos.org/plosntds/article?id=10.1371/journal.pntd.0004021. doi: 10.1371/journal.pntd.0004021 26291988 PMC4546333

[33] Nausch N, Dawson EM, Midzi N, Mduluza T, Mutapi F, Doenhoff MJ. Field evaluation of a new antibody-based diagnostic for Schistosoma haematobium and S. mansoni at the point-of-care in northeast Zimbabwe. BMC Infect Dis [Internet]. 2014 Mar 26 [cited 2023 May 27];14(1):1–9. Available from: https://bmcinfectdis.biomedcentral.com/articles/10.1186/1471-2334-14-165. doi: 10.1186/1471-2334-14-165 24666689 PMC4021455

[34] Bansal GP, Vengesai A, Cao Y, Mduluza T, Kumar N. Antibodies elicited during natural infection in a predominantly Plasmodium falciparum transmission area cross-react with sexual stage-specific antigen in P. vivax. Acta Trop [Internet]. 2017 Jun 1 [cited 2020 Aug 4];170:105–11. Available from: https://pubmed.ncbi.nlm.nih.gov/28257812/. doi: 10.1016/j.actatropica.2017.02.032 28257812 PMC5410398

[35] Fatima A, Abdelaali B, Corstjens PLAM, Abderrahim S, El Bachir A, Mohamed R. Survey and Diagnostic Challenges after Transmission-Stop: Confirming Elimination of Schistosomiasis haematobium in Morocco. J Parasitol Res [Internet]. 2020 [cited 2024 May 29];2020. Available from: /pmc/articles/PMC7212323/. doi: 10.1155/2020/9705358 32411424 PMC7212323

[36] de Souza DK, Otchere J, Sumboh JG, Asiedu O, Opare J, Asemanyi-Mensah K, et al. Finding and eliminating the reservoirs: Engage and treat, and test and treat strategies for lymphatic filariasis programs to overcome endgame challenges. Front Trop Dis. 2022 Aug 11;3:953094.

[37] Gruninger SK, Rasamoelina T, Rakotoarivelo RA, Razafindrakoto AR, Rasolojaona ZT, Rakotozafy RM, et al. Prevalence and risk distribution of schistosomiasis among adults in Madagascar: a cross-sectional study. Infect Dis Poverty [Internet]. 2023 Dec 1 [cited 2023 Dec 15];12(1):1–10. Available from: https://idpjournal.biomedcentral.com/articles/10.1186/s40249-023-01094-z 37098581 10.1186/s40249-023-01094-z PMC10127445

[38] Tokarz R, Mishra N, Tagliafierro T, Sameroff S, Caciula A, Chauhan L, et al. A multiplex serologic platform for diagnosis of tick-borne diseases. Sci Rep [Internet]. 2018 Dec 1 [cited 2020 Nov 23];8(1). Available from: https://pubmed-ncbi-nlm-nih-gov.ukzn.idm.oclc.org/29453420/. doi: 10.1038/s41598-018-21349-2 29453420 PMC5816631

[39] Sachse K, Rahman KS, Schnee C, Müller E, Peisker M, Schumacher T, et al. A novel synthetic peptide microarray assay detects Chlamydia species-specific antibodies in animal and human sera. Sci Rep [Internet]. 2018 Dec 1 [cited 2020 Nov 24];8(1). Available from: https://pubmed-ncbi-nlm-nih-gov.ukzn.idm.oclc.org/29549361/. doi: 10.1038/s41598-018-23118-7 29549361 PMC5856796

[40] Maksimov P, Zerweck J, Maksimov A, Hotop A, Groß U, Pleyer U, et al. Peptide microarray analysis of in silico-predicted epitopes for serological diagnosis of Toxoplasma gondii infection in humans. Clin Vaccine Immunol [Internet]. 2012 Jun [cited 2020 Nov 22];19(6):865–74. Available from: https://pubmed-ncbi-nlm-nih-gov.ukzn.idm.oclc.org/22496494/. doi: 10.1128/CVI.00119-12 22496494 PMC3370440

[41] Torlakovic EE, Francis G, Garratt J, Gilks B, Hyjek E, Ibrahim M, et al. Standardization of negative controls in diagnostic immunohistochemistry: Recommendations from the international Ad Hoc expert panel. Appl Immunohistochem Mol Morphol [Internet]. 2014 [cited 2022 May 10];22(4):241–52. Available from: https://journals.lww.com/appliedimmunohist/Fulltext/2014/04000/Standardization_of_Negative_Controls_in_Diagnostic.1.aspx. doi: 10.1097/PAI.0000000000000069 24714041 PMC4206554

[42] West R, Kobokovich A. Understanding the Accuracy of Diagnostic and Serology Tests: Sensitivity and Specificity Factsheet. 2020;

[43] Magalhães F do C, Moreira JMP, de Rezende MC, Favero V, Graeff-Teixeira C, Coelho PMZ, et al. Evaluation of isotype-based serology for diagnosis of Schistosoma mansoni infection in individuals living in endemic areas with low parasite burden. Acta Trop. 2023 Dec 1;248. doi: 10.1016/j.actatropica.2023.107017 37774894

[44] De Benedetti S, Di Pisa F, Fassi EMA, Cretich M, Musicò A, Frigerio R, et al. Structure, Immunoreactivity, and In Silico Epitope Determination of SmSPI S. mansoni Serpin for Immunodiagnostic Application. Vaccines 2021, Vol 9, Page 322 [Internet]. 2021 Apr 1 [cited 2022 May 24];9(4):322. Available from: https://www.mdpi.com/2076-393X/9/4/322/htm. 33915716 10.3390/vaccines9040322 PMC8066017

[45] Diagnostic target product profiles for monitoring, evaluation and surveillance of schistosomiasis control programmes [Internet]. [cited 2022 Jan 28]. Available from: https://www.who.int/publications/i/item/9789240031104.

[46] Ogongo P. Identification and evaluation of Schistosoma mansoni proteins as diagnostic targets for schistosomiasis. Int J Infect Dis. 2014 Apr 1;21:365.

[47] Schistosomiasis [Internet]. [cited 2023 Dec 4]. Available from: https://www.who.int/news-room/fact-sheets/detail/schistosomiasis.

[48] Rivera J, Mu Y, Gordon CA, Jones MK, Cheng G, Cai P. Current and upcoming point-of-care diagnostics for schistosomiasis. Trends Parasitol [Internet]. 2023 Nov 23 [cited 2023 Dec 4]; Available from: https://linkinghub.elsevier.com/retrieve/pii/S1471492223002805. doi: 10.1016/j.pt.2023.10.005 38000956

[49] Lagatie O, Granjon E, Odiere MR, Zrein M, Stuyver LJ. Assessment of multiplex Onchocerca volvulus peptide ELISA in non-endemic tropical regions. Parasites and Vectors [Internet]. 2019 Nov 29 [cited 2024 May 29];12(1):1–8. Available from: https://parasitesandvectors.biomedcentral.com/articles/10.1186/s13071-019-3824-x. 31783767 10.1186/s13071-019-3824-x PMC6884800

