# Supplementary material for: Identification of Schistosoma haematobium and Schistosoma mansoni linear B-cell epitopes with diagnostic potential using in silico immunoinformatic tools and peptide microarray technology
Source: PLoS Negl Trop Dis. 2024 Aug 22;18(8):e0011887. doi: 10.1371/journal.pntd.0011887 (PMC11373837; doi:10.1371/journal.pntd.0011887)
Supplement: S1 File — (PDF) [file pntd.0011887.s001.pdf]

Search Strategy

Schistosomiasis or bilharzia or snail fever or schistosome or schistosoma or S. haematobium or S. mansoni or bilharziasis or katayama fever or Bilharziase.

AND

Peptides or Epitopes or antigens or proteins or polypeptides or antigenic determinant

PubMed Search Results (February 14 2022)

Query	Results
(Schistosomiasis[Title/Abstract] OR bilharzia[Title/Abstract] OR snail fever[Title/Abstract] OR schistosome[Title/Abstract] OR schistosoma[Title/Abstract] OR S. haematobium[Title/Abstract] OR S. mansoni[Title/Abstract] OR bilharziasis[Title/Abstract] OR katayama fever[Title/Abstract] OR Bilharziase[Title/Abstract]) AND (Peptides[Title/Abstract] OR Epitopes[Title/Abstract] OR antigens[Title/Abstract] OR proteins[Title/Abstract] OR polypeptides[Title/Abstract] OR antigenic determinant[Title/Abstract])	3,893
Peptides[Title/Abstract] OR Epitopes[Title/Abstract] OR antigens[Title/Abstract] OR proteins[Title/Abstract] OR polypeptides[Title/Abstract] OR antigenic determinant[Title/Abstract]	1,671,179
Schistosomiasis[Title/Abstract] OR bilharzia[Title/Abstract] OR snail fever[Title/Abstract] OR schistosome[Title/Abstract] OR schistosoma[Title/Abstract] OR S. haematobium[Title/Abstract] OR S. mansoni[Title/Abstract] OR bilharziasis[Title/Abstract] OR katayama fever[Title/Abstract] OR Bilharziase[Title/Abstract]	32,382

CINAHL (February 16 2022)

#	Query	Limiters/Expanders	Last Run Via	Results
S21	S11 AND S20	Search modes - Find all my search terms	Interface - EBSCOhost Research Databases Search Screen - Advanced Search Database - CINAHL	143
S20	S13 OR S14 OR S15 OR S16 OR S17 OR S18 OR S19	Search modes - Find all my search terms	Interface - EBSCOhost Research Databases Search Screen - Advanced Search Database - CINAHL	180,318
S19	AB Antigenic determinants	Search modes - Find all my search terms	Interface - EBSCOhost Research Databases Search Screen - Advanced Search Database - CINAHL	75
S18	AB Polypeptides	Search modes - Find all my search terms	Interface - EBSCOhost Research Databases Search Screen - Advanced Search Database - CINAHL	1,953
S17	AB Recombinant protein	Search modes - Find all my search terms	Interface - EBSCOhost Research Databases Search Screen - Advanced Search Database - CINAHL	3,772
S16	AB Recombinant antigen	Search modes - Find all my search terms	Interface - EBSCOhost Research Databases Search Screen - Advanced Search Database - CINAHL	959
S15	AB Proteins	Search modes - Find all my search terms	Interface - EBSCOhost Research Databases Search Screen - Advanced Search Database - CINAHL	153,202
S14	AB Antigens	Search modes - Find all my search terms	Interface - EBSCOhost Research Databases Search Screen - Advanced Search Database - CINAHL	30,058
S13	AB Epitopes	Search modes - Find all my search terms	Interface - EBSCOhost Research Databases Search Screen - Advanced Search Database - CINAHL	2,457

S12	AB Peptides	Search modes - Find all my search terms	Interface - EBSCOhost Research Databases Search Screen - Advanced Search Database - CINAHL	23,650
S11	S1 OR S2 OR S3 OR S4 OR S5 OR S6 OR S7 OR S8 OR S9 OR S10	Search modes - Find all my search terms	Interface - EBSCOhost Research Databases Search Screen - Advanced Search Database - CINAHL	1,012
S10	AB Katayama fever	Search modes - Find all my search terms	Interface - EBSCOhost Research Databases Search Screen - Advanced Search Database - CINAHL	4
S9	AB Bilharziase	Search modes - Find all my search terms	Interface - EBSCOhost Research Databases Search Screen - Advanced Search Database - CINAHL	0
S8	AB Bilharziasis	Search modes - Find all my search terms	Interface - EBSCOhost Research Databases Search Screen - Advanced Search Database - CINAHL	17
S7	AB S. mansoni	Search modes - Find all my search terms	Interface - EBSCOhost Research Databases Search Screen - Advanced Search Database - CINAHL	202
S6	AB S. haematobium	Search modes - Find all my search terms	Interface - EBSCOhost Research Databases Search Screen - Advanced Search Database - CINAHL	122
S5	AB Schistosoma	Search modes - Find all my search terms	Interface - EBSCOhost Research Databases Search Screen - Advanced Search Database - CINAHL	532
S4	AB Schistosome	Search modes - Find all my search terms	Interface - EBSCOhost Research Databases Search Screen - Advanced Search Database - CINAHL	145
S3	AB Snail fever	Search modes - Find all my search terms	Interface - EBSCOhost Research Databases Search Screen - Advanced Search Database - CINAHL	6

S2	AB Bilharzia	Search modes - Find all my search terms	Interface - EBSCOhost Research Databases Search Screen - Advanced Search Database - CINAHL	25
S1	AB Schistosomiasis	Search modes - Find all my search terms	Interface - EBSCOhost Research Databases Search Screen - Advanced Search Database - CINAHL	778

Cochrane Library (February 20 2022)

105 Trials matching Schistosomiasis or bilharzia or snail fever or schistosome or schistosoma or S. haematobium or S. mansoni or bilharziasis or katayama fever or Bilharziase. in Title Abstract Keyword AND Peptides or Epitopes or antigens or proteins or polypeptides or antigenic determinant in Title Abstract Keyword - (Word variations have been searched)

Cochrane Central Register of Controlled Trials

Issue 2 of 12, February 2022

105 Trials matching **Schistosomiasis or bilharzia or snail fever or schistosome or schistosoma or S. haematobium or S. mansoni or bilharziasis or katayama fever or Bilharziase. in Title Abstract Keyword AND Peptides or Epitopes or antigens or proteins or polypeptides or antigenic determinant in Title Abstract Keyword - (Word variations have been searched)**

Cochrane Central Register of Controlled Trials
Issue 2 of 12, February 2022

PsycInfo (February 20 2022)

#	Query	Limiters/Expanders	Last Run Via	Results
S21	S11 AND S20	Search modes - Find all my search terms	Interface - EBSCOhost Research Databases Search Screen - Advanced Search Database - APA PsycInfo	16
S20	S12 OR S13 OR S14 OR S15 OR S16 OR S17 OR S18 OR S19	Search modes - Find all my search terms	Interface - EBSCOhost Research Databases Search Screen - Advanced Search Database - APA PsycInfo	96,649
S19	AB antigenic determinant	Search modes - Boolean/Phrase	Interface - EBSCOhost Research Databases Search Screen - Advanced Search Database - APA PsycInfo	9
S18	AB recombinants protein	Search modes - Boolean/Phrase	Interface - EBSCOhost Research Databases Search Screen - Advanced Search Database - APA PsycInfo	337
S17	AB polypeptides	Search modes - Boolean/Phrase	Interface - EBSCOhost Research Databases Search Screen - Advanced Search Database - APA PsycInfo	1,300
S16	AB Proteins	Search modes - Boolean/Phrase	Interface - EBSCOhost Research Databases Search Screen - Advanced Search Database - APA PsycInfo	81,707
S15	AB Antigens	Search modes - Boolean/Phrase	Interface - EBSCOhost Research Databases Search Screen - Advanced Search Database - APA PsycInfo	4,623
S14	AB Epitome	Search modes - Boolean/Phrase	Interface - EBSCOhost Research Databases Search Screen - Advanced Search Database - APA PsycInfo	242
S13	AB Epitopes	Search modes - Boolean/Phrase	Interface - EBSCOhost Research Databases Search Screen - Advanced Search Database - APA PsycInfo	893
S12	AB Peptides	Search modes - Boolean/Phrase	Interface - EBSCOhost Research Databases	15,759

			Search Screen - Advanced Search Database - APA PsycInfo	
S11	S1 OR S2 OR S3 OR S4 OR S5 OR S6 OR S7 OR S8 OR S9 OR S10	Search modes - Find all my search terms	Interface - EBSCOhost Research Databases Search Screen - Advanced Search Database - APA PsycInfo	150
S10	AB Katayama fever	Search modes - SmartText Searching	Interface - EBSCOhost Research Databases Search Screen - Advanced Search Database - APA PsycInfo	1
S9	AB Katayama fever	Search modes - Find all my search terms	Interface - EBSCOhost Research Databases Search Screen - Advanced Search Database - APA PsycInfo	0
S8	AB Bilharziasis	Search modes - Find all my search terms	Interface - EBSCOhost Research Databases Search Screen - Advanced Search Database - APA PsycInfo	1
S7	AB S. mansoni	Search modes - Find all my search terms	Interface - EBSCOhost Research Databases Search Screen - Advanced Search Database - APA PsycInfo	16
S6	AB S. haematobium	Search modes - Find all my search terms	Interface - EBSCOhost Research Databases Search Screen - Advanced Search Database - APA PsycInfo	5
S5	AB schistosome	Search modes - Find all my search terms	Interface - EBSCOhost Research Databases Search Screen - Advanced Search Database - APA PsycInfo	11
S4	AB schistosoma	Search modes - Find all my search terms	Interface - EBSCOhost Research Databases Search Screen - Advanced Search Database - APA PsycInfo	42
S3	AB Snail fever	Search modes - Find all my search terms	Interface - EBSCOhost Research Databases Search Screen - Advanced Search Database - APA PsycInfo	1
S2	AB bilharzia	Search modes - Find all my search terms	Interface - EBSCOhost Research Databases	1

			Search Screen - Advanced Search Database - APA PsycInfo	
S1	AB Schistosomiasis	Search modes - Find all my search terms	Interface - EBSCOhost Research Databases Search Screen - Advanced Search Database - APA PsycInfo	96

EMBASE

**20**	**limit 19 to yr="2000 - 2022"**	**2239**	**Type**
**19**	**10 and 18**	**3839**	**Advanced**
**18**	**11 or 12 or 13 or 14 or 15 or 16 or 17**	**1810167**	**Advanced**
**17**	**antigenic determinants.ab.**	**6324**	**Advanced**
**16**	**recombinant proteins.ab.**	**16364**	**Advanced**
**15**	**polypeptides.ab.**	**38960**	**Advanced**
**14**	**proteins.ab.**	**1376927**	**Advanced**
**13**	**Antigens.ab.**	**227996**	**Advanced**
**12**	**Epitopes.ab.**	**62297**	**Advanced**
**11**	**Peptides.ab.**	**261362**	**Advanced**
**10**	**1 or 2 or 3 or 4 or 5 or 6 or 7 or 8 or 9**	**25428**	**Advanced**
**9**	**Katayama fever.ab.**	**39**	**Advanced**
**8**	**Bilharziasis.ab.**	**446**	**Advanced**
**7**	**S mansoni.ab.**	**6163**	**Advanced**
**6**	**S haematobium.ab.**	**2137**	**Advanced**
**5**	**Schistosoma.ab.**	**16246**	**Advanced**
**4**	**Schistosome.ab.**	**4700**	**Advanced**
**3**	**Snail fever.ab.**	**14**	**Advanced**
**2**	**Bilharzia.ab.**	**272**	**Advanced**
**1**	**Schistosomiasis.ab.**	**14550**	**Advanced**

medRxiv preprint server for health sciences.

246 Results for term "schistosomiasis and proteins "
