# Supplementary material for: Identification of Schistosoma haematobium and Schistosoma mansoni linear B-cell epitopes with diagnostic potential using in silico immunoinformatic tools and peptide microarray technology
Source: PLoS Negl Trop Dis. 2024 Aug 22;18(8):e0011887. doi: 10.1371/journal.pntd.0011887 (PMC11373837; doi:10.1371/journal.pntd.0011887)
Supplement: S3 File — (PDF) [file pntd.0011887.s003.pdf]

S. haematobium						
**Antigen (accession)**	**Description**	**Protein family**	Serological assay	**Sensitivity**	**Specificity**	**AUC& (95% CI)**
MS3_10385	neuroserpin+	Serpin	Serum IgG protein microarray	-	-	0.88 (0.83-0.92)
MS3_10186	IPSE	None predicted	Serum IgG protein microarray	-	-	0.88 (0.83-0.92)
MS3_06193	PUR-alpha-like protein	PUR DNA binding protein (uniport.org)	Serum IgG protein microarray	-	-	0.71 (0.64-0.79)
MS3_01466	band 7 protein	Mec 2 (uniport.org)	Serum IgG protein microarray	-	-	0.69 (0.62-0.75)
MS3_05950	16 kDa calcium-binding protein	None predicted	Serum IgG protein microarray	-	-	0.76 (0.70-0.82)
MS3_09198	CD63 antigen+	Tetraspanin (uniport.org)	Serum IgG protein microarray	-	-	0.79 (0.73-0.85)
MS3_09779	cathepsin B-like peptidase (C01 family) +	Peptidase C1 (uniport.org)	Serum IgG protein microarray	-	-	0.84 (0.76-0.93)
MS3_07972	ferritin, heavy polypeptide 1+	Ferritin (uniport.org)	Serum IgG protein microarray	-	-	0.86 (0.80-0.92)
MS3_09207	hemoglobinase (C13 family) +	Peptidase C13 (uniport.org)	Serum IgG protein microarray	-	-	0.78 (0.71-0.84)
MS3_01370	CD63 antigen+	Tetraspanin	Serum IgG protein microarray	-	-	0.78 (0.71-0.84)
MS3_10385	neuroserpin	Serpin	Urine IgG protein microarray	-	-	0.93 (0.85-1.00)
MS3_10186	IPSE	None predicted	Urine IgG protein microarray	-	-	0.88 (0.80-0.97)
MS3_06193	PUR-alpha-like protein	PUR DNA binding (uniport.org)	Urine IgG protein microarray	-	-	0.83 (0.75-0.91)
MS3_01466	band 7 protein	Mec 2 (uniport.org)	Urine IgG protein microarray	-	-	0.75 (0.66-0.84)
MS3_05950	16 kDa calcium-binding protein	None predicted (uniport.org)	Urine IgG protein microarray	-	-	0.72 (0.62-0.82)
MS3_09198	CD63 antigen+	Tetraspain	Urine IgG protein microarray	-	-	0.83 (0.73-0.92)
MS3_09779	cathepsin B-like peptidase (C01 family) +	Peptidase C1 (uniport.org)	Urine IgG protein microarray	-	-	0.66 (0.55-0.76)

MS3_07972	ferritin, heavy polypeptide 1+	Ferritin (uniport.org)	Urine IgG protein microarray	-	-	0.65 (0.54-0.76)
MS3_09207	hemoglobinase (C13 family) +	Haemoglobinase (uniport.org)	Urine IgG protein microarray	-	-	0.68 (0.57-0.78)
MS3_01370	CD63 antigen	Tetraspanin (uniport.org)	Urine IgG protein microarray	-	-	0.66 (0.56-0.77)
MS3_10385	Neuroserpin+	Serpin	Serum IgG ELISA	-	-	0.80 (0.70-0.91)
MS3_10186	IPSE	Tetraspsnin	Serum IgG ELISA	-	-	0.88 (0.82-0.94)
MS3_09198	CD63 antigen+	Tetraspanin (uniport.org)	Serum IgG ELISA	-	-	0.82 (0.74-0.91)
MS3_01370	CD63 antigen	Tetraspsnin	Serum IgG ELISA	-	-	0.93 (0.89-0.97)
Sh-TSP2		Tetraspanin	Serum IgG ELISA	-	-	0.98 (0.95-1.00)
MS3_10385	Neuroserpin+	Serpin	Urine IgG ELISA	-	-	0.78 (0.71-0.86)
MS3_10186	IPSE	Tetraspanin	Urine IgG ELISA	-	-	0.69 (0.62-0.77)
MS3_09198	CD63 antigen+	Tetraspanin (uniport.org)	Urine IgG ELISA	-	-	0.78 (0.67-0.88)
MS3_01370	CD63 antigen	Tetraspanin	Urine IgG ELISA	-	-	0.81 (0.72-0.89)
Sh-TSP2	Tetraspanin	Tetraspanin	Urine IgG ELISA	-	-	0.96 (0.93-0.99)
MS3_01370	CD63 antigen	Tetraspanin	Serum IgG POC-ICTs	89 %	100 %	-
Sh-TSP2	Tetraspanins	Tetraspanin	Serum IgG POC-ICTs	75 %	100 %	-
Sh-TPS-4	Tetraspanins	Tetraspanin	Urine IgG ELISA	-	-	0.87
Sh-TPS-5	Tetraspanins	Tetraspanin	Urine IgG ELISA	-	-	0.93
Sh-TPS-18	Tetraspanins	Tetraspanin	Urine IgG ELISA	-	-	0.88
AAA19730	Serine protease inhibitor	Serpin	Serum IgG Luminex immunoassay	92.5 %	90 %	0.947
	Sm 25		Serum IgG Luminex immunoassay	-	-	0.711
S. mansoni						
**Antigen (accession)**	**Description**	**Protein family**	Serological assay	**Sensitivity**	**Specificity**	**AUC& (95% CI)**
Smp_049250.1	Major egg antigen	Cheparone	Serum IgG ELISA	87.1 (95 % CI 78.55-93.15 %)	89.09 % (77.75-95.89 %)	-

Smp_138060 - CCD60408.1	MEG 3.2	MEG 3	Serum IgM ELISA	90 %	70 %	-
Smp_138060 - CCD60408.1	MEG 3.2	MEG 3	Serum IgG ELISA	90 %	83 %	-
Smp_138090 - CAZ30619.1)	MEG 3.4	MEG 3	Serum IgM ELISA	75 %	90 %	-
SmSPI	Serine protease inhibitor	Serpin	Serum IgG protein microarray	91.7 %	93.3 %	-
SmSPI	Serine protease inhibitor	Serpin	Serum IgG DELFIA	83.7 %	61.4 %	-
AAB81008	RP 26	SAPLIP	Serum IgG ELISA	74.5 % (95 % CI 66.7-82.8 %)	57.4 % (95 % CI 47.2-67.2)	-
CCD60071	Serine protease inhibitor	Serpin	Serum IgG ELISA	65.3 % (95 % CI 55.0-74.6 %)	62.4 % (52.2-71.8 %)	-
Sm CRT	Calreticulin	Calreticulin	Serum IgG ELISA	89.7 %	100 %	-
AAB81008	RP 26 (Sm 22.3)	SAPLIP	Serum IgG Luminex immunoassay	67.8 %	89.5 %	0.833
CCD60071	Serine protease inhibitor	Serpin	Serum IgG Luminex immunoassay	89.4 %	81.5 %	0.888
	Major egg antigen (ME)	Cheparone	Serum IgG Luminex immunoassay	-	-	0.746
	Sm 25		Serum IgG Luminex immunoassay	-	-	0.741
	RP 26	SAPLIP	Serum IgG western blot	100 %	100 %	-
	RP 26	SAPLIP	Serum IgG western blot	89 %	-	-
