# Supplementary material for: Identification of Schistosoma haematobium and Schistosoma mansoni linear B-cell epitopes with diagnostic potential using in silico immunoinformatic tools and peptide microarray technology
Source: PLoS Negl Trop Dis. 2024 Aug 22;18(8):e0011887. doi: 10.1371/journal.pntd.0011887 (PMC11373837; doi:10.1371/journal.pntd.0011887)
Supplement: S5 File — Receiver operating characteristics (ROC) curve and area under the ROC curve (AUC) to detect S. mansoni (a-q) and S. haematobium (r-t) patient serum IgG. (PDF) [file pntd.0011887.s005.pdf]

Diagnostic performance of peptides to detect *S. mansoni* patient IgG.

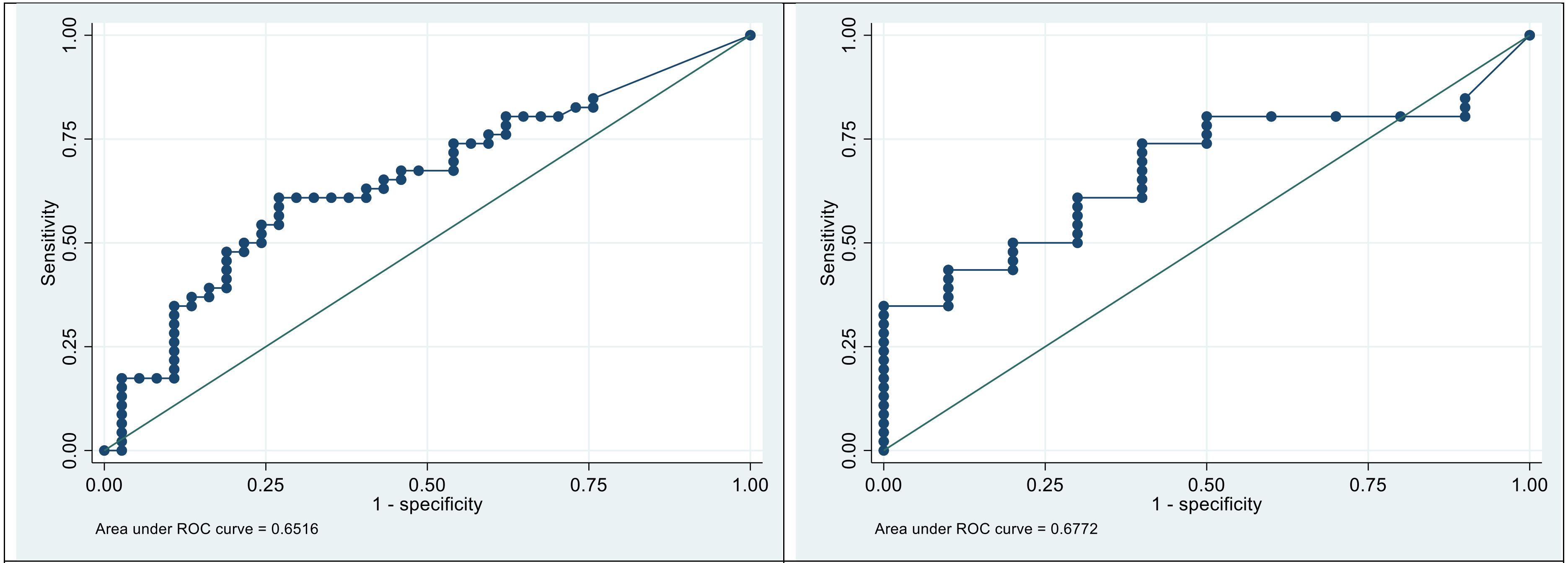

S. mansoni positives versus *S. mansoni* negatives	*S. mansoni* positive versus healthy controls
**Peptide MS3_10186-119-35**	

a

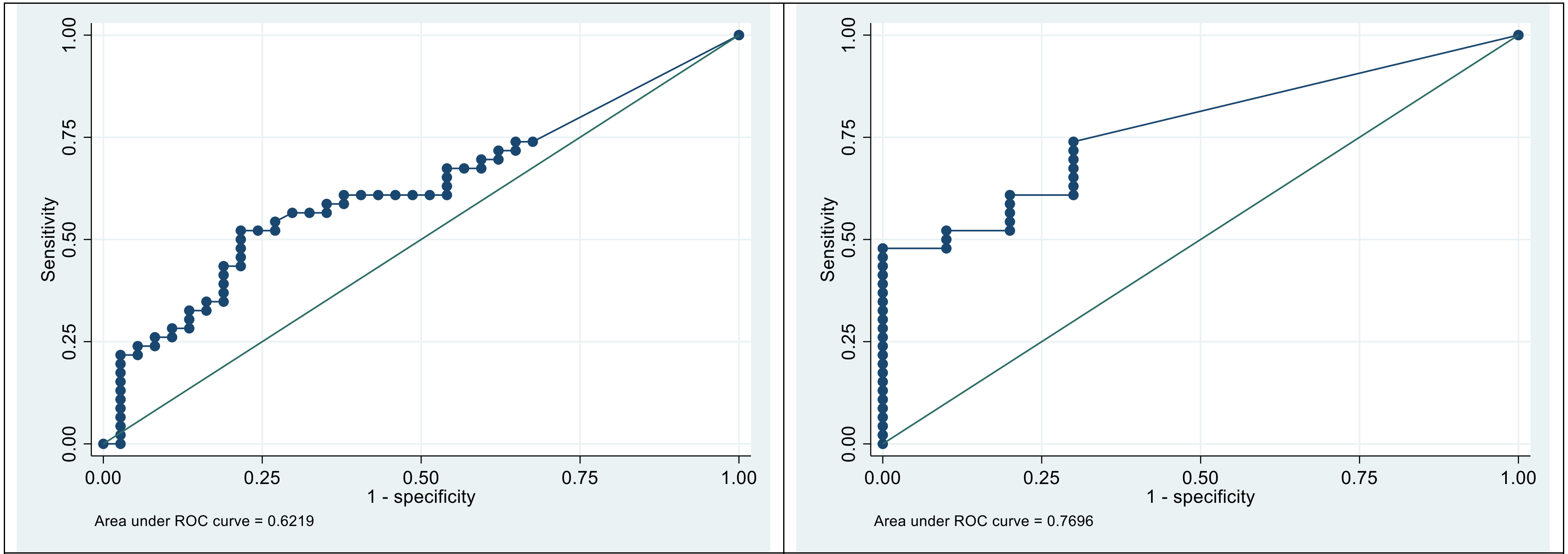

S. mansoni positives versus *S. mansoni* negatives	*S. mansoni* positive versus healthy controls
**Peptide MS3_10186-123-131**	

b

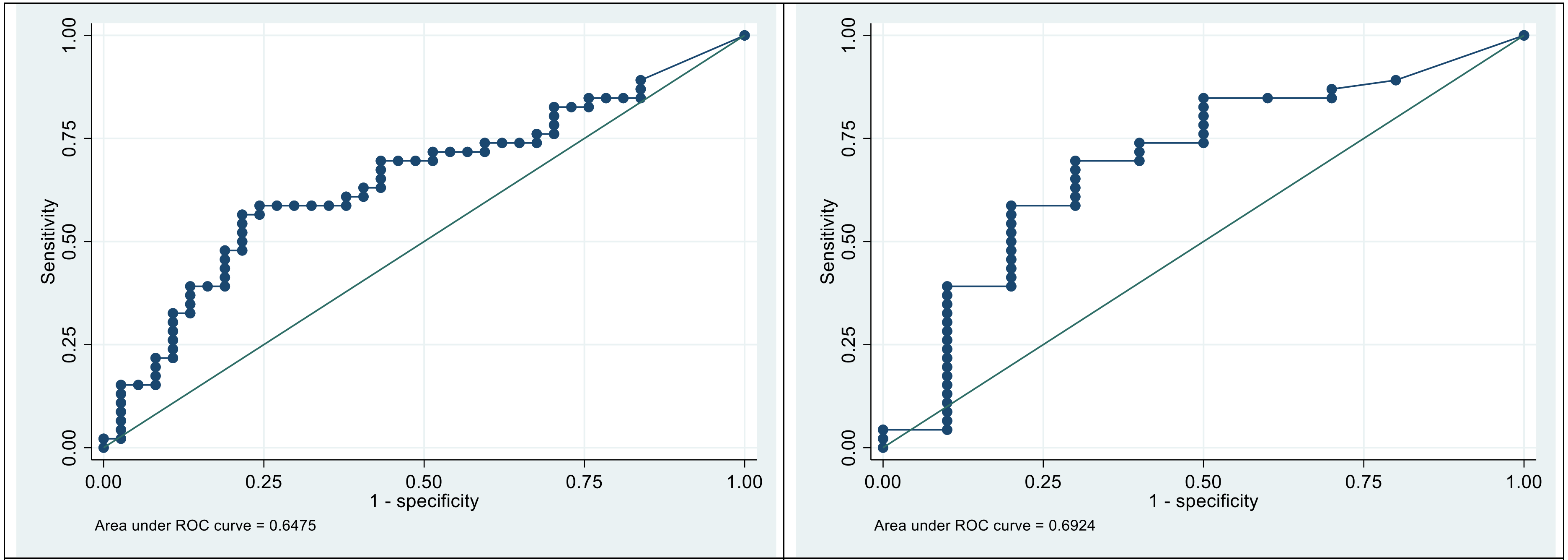

S. mansoni positives versus *S. mansoni* negatives	*S. mansoni* positive versus healthy controls

Peptide MS3_10385-278-291

c

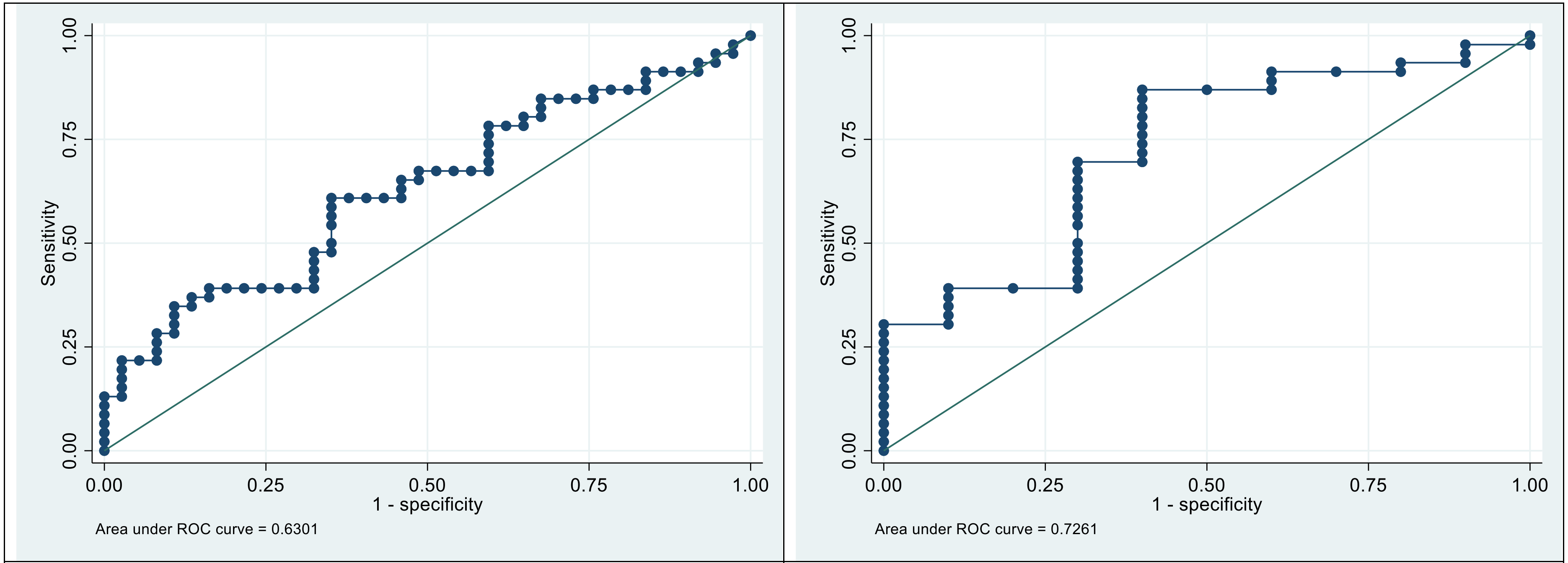

S. mansoni positives versus *S. mansoni* negatives	*S. mansoni* positive versus healthy controls
**Peptide MS3_10385-339-354**	

d

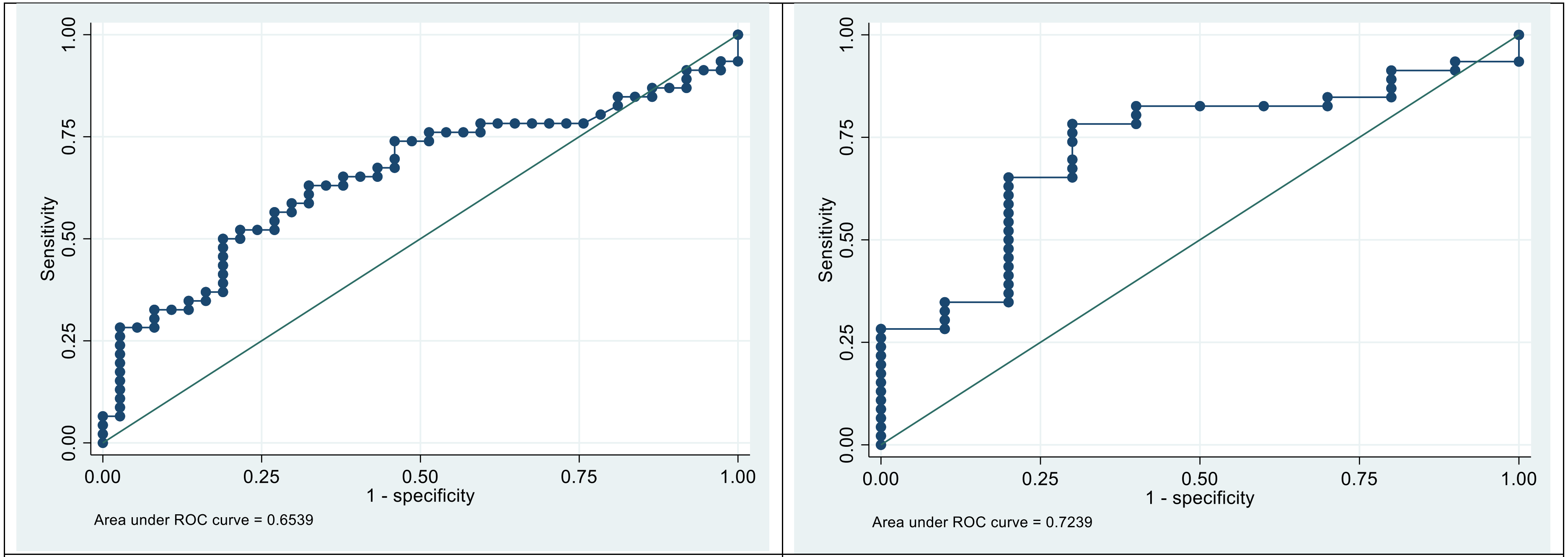

S. mansoni positives versus *S. mansoni* negatives	*S. mansoni* positive versus healthy controls

Peptide SmSPI-177-193

e

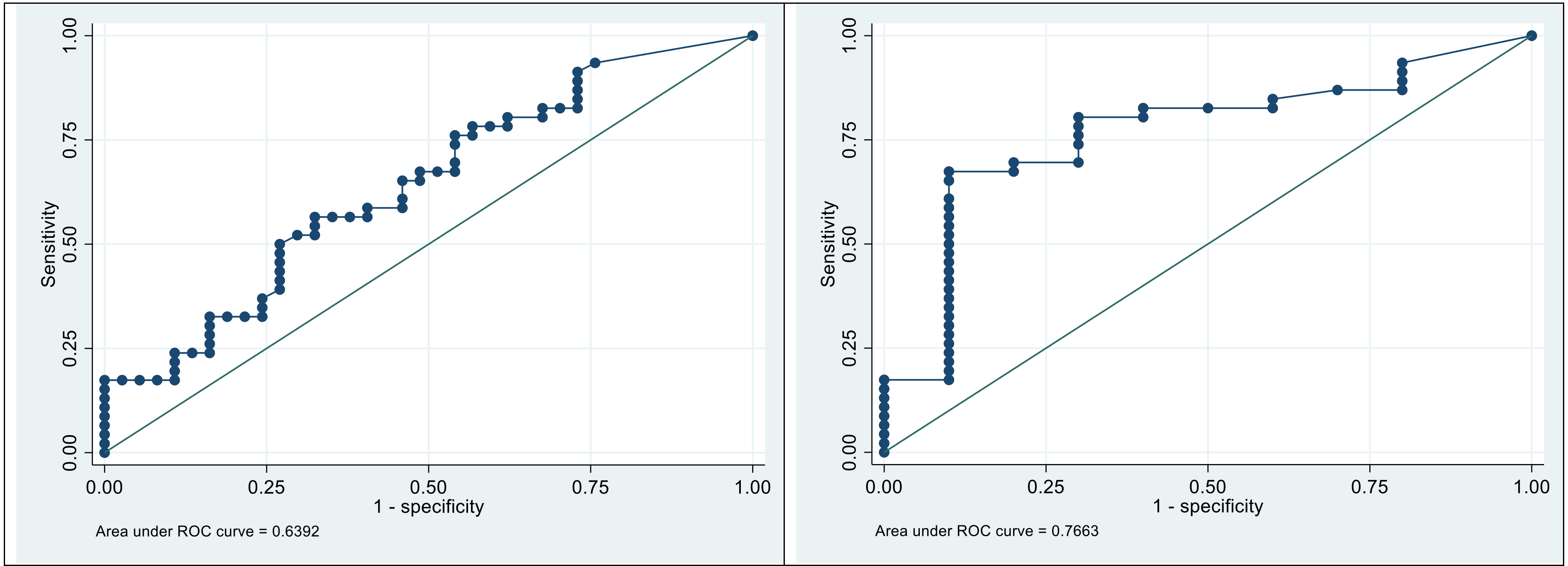

S. mansoni positives versus *S. mansoni* negatives	*S. mansoni* positive versus healthy controls
**Peptide SmSPI-378-388**	

f

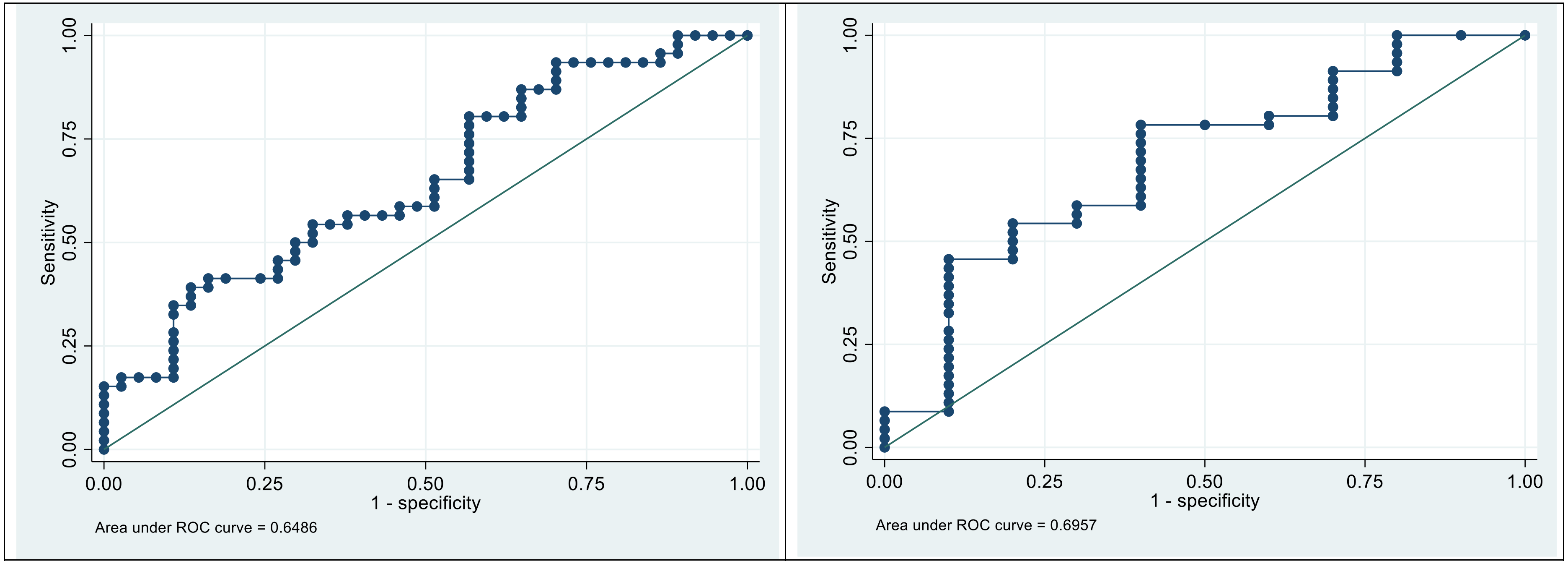

S. mansoni positives versus *S. mansoni* negatives	*S. mansoni* positives versus healthy controls

Peptide SmSPI-362-378

h

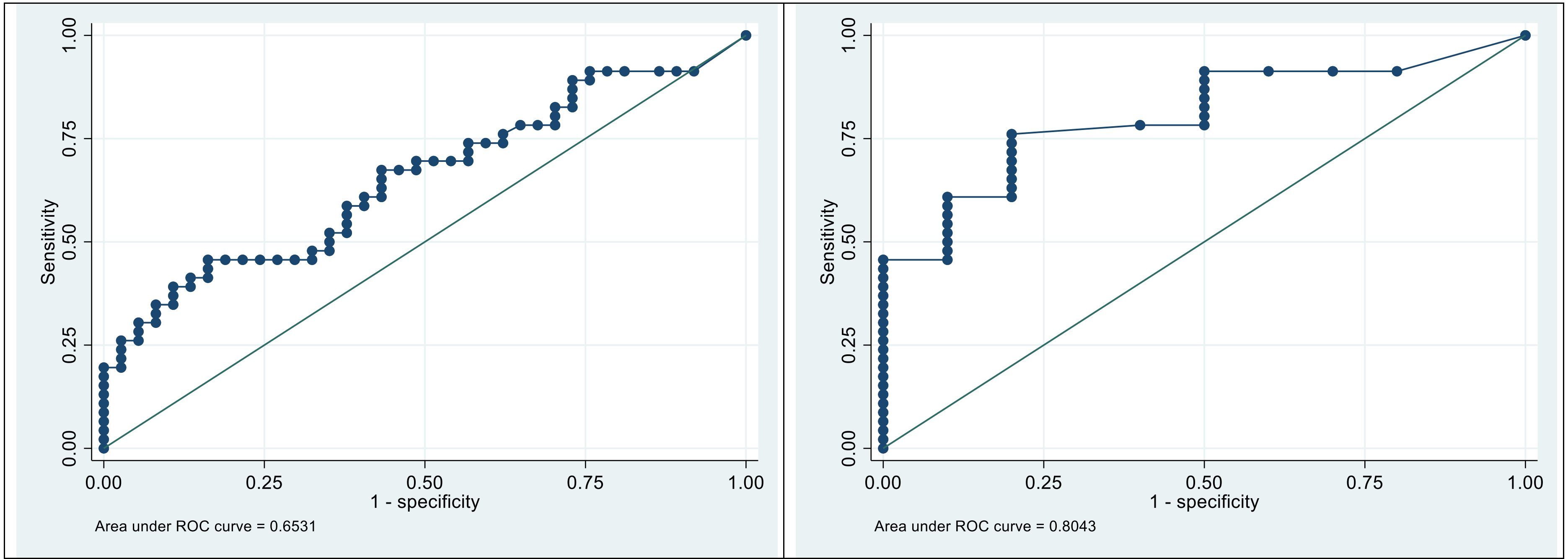

S. mansoni positives versus *S. mansoni* negatives | *S. mansoni* positives versus healthy controls

Peptide AAB81008-19-30

i

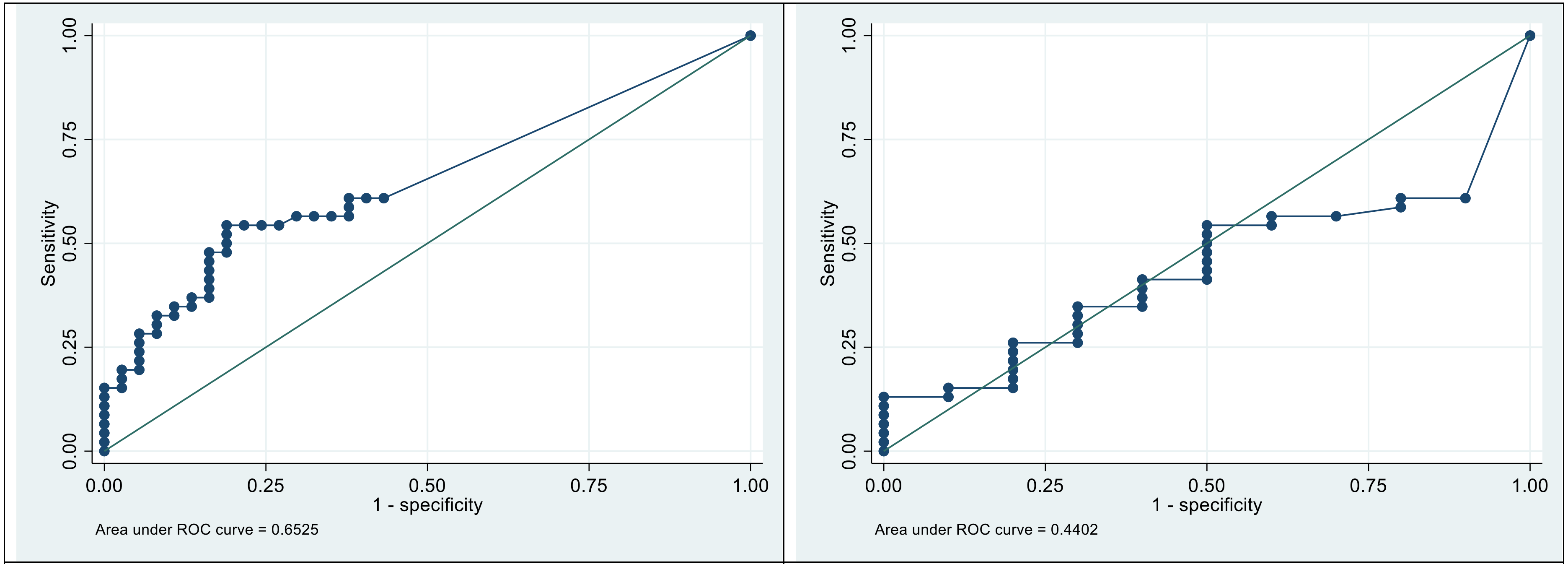

S. mansoni positives versus *S. mansoni* negatives	*S. mansoni* positives versus healthy controls

Peptide SmSP1-310-323

j

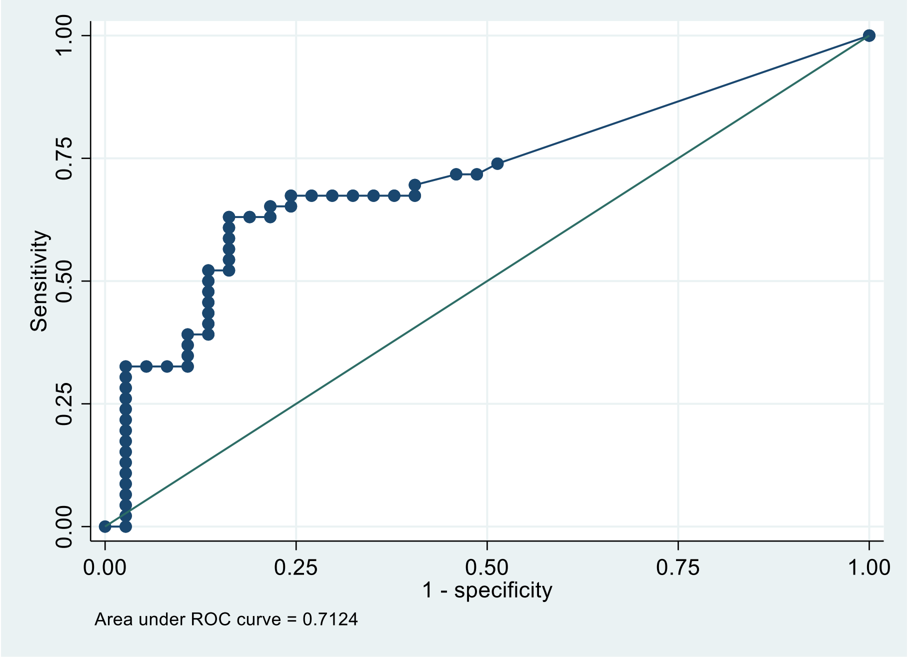

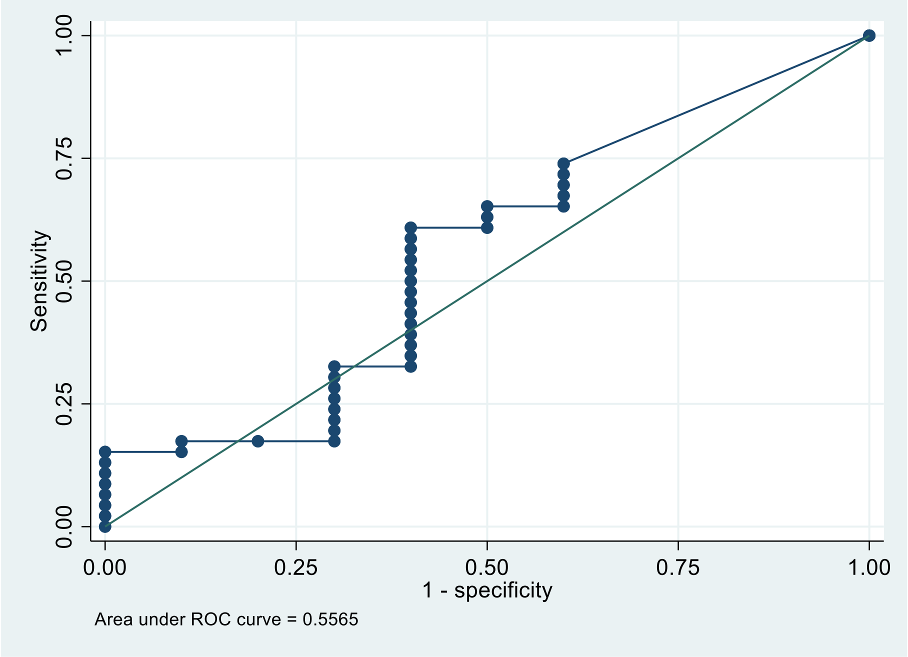

S. mansoni positives versus *S. mansoni* negatives	*S. mansoni* positives versus healthy controls
**Peptide SmSP1_359_372**	

k

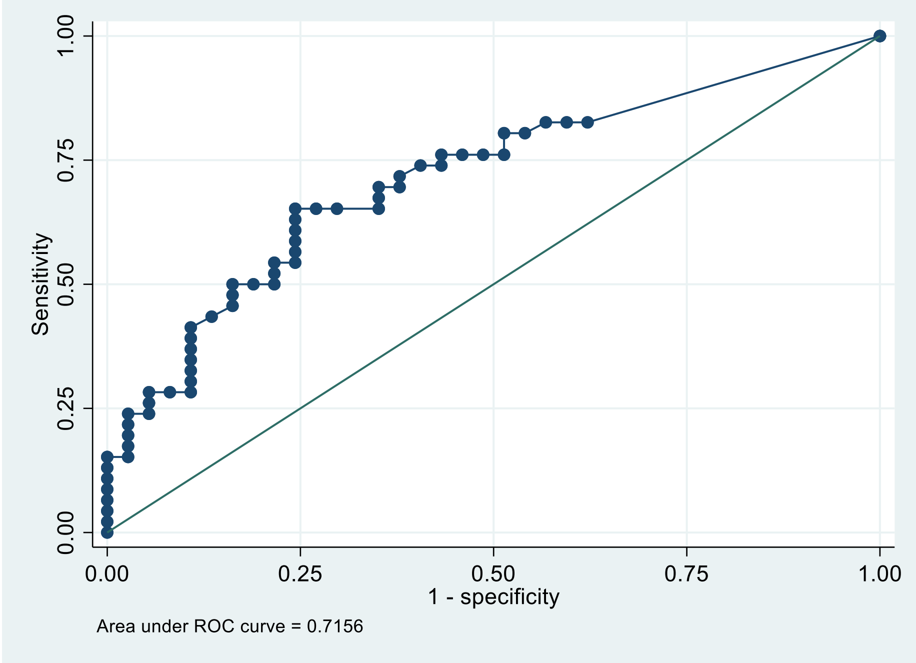

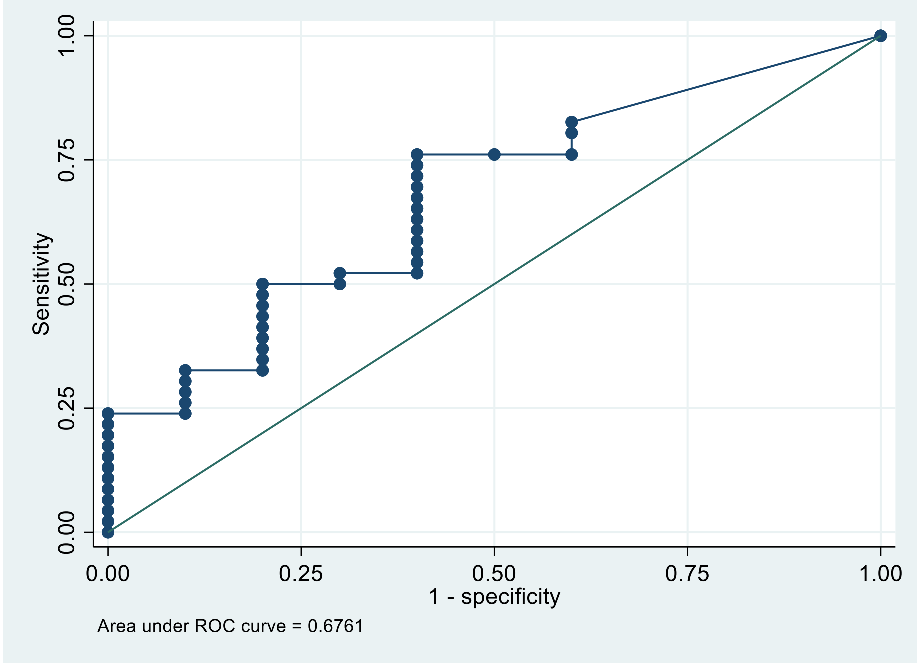

S. mansoni positives versus *S. mansoni* negatives	*S. mansoni* positives versus healthy controls

Peptide Smp 126160-438-452

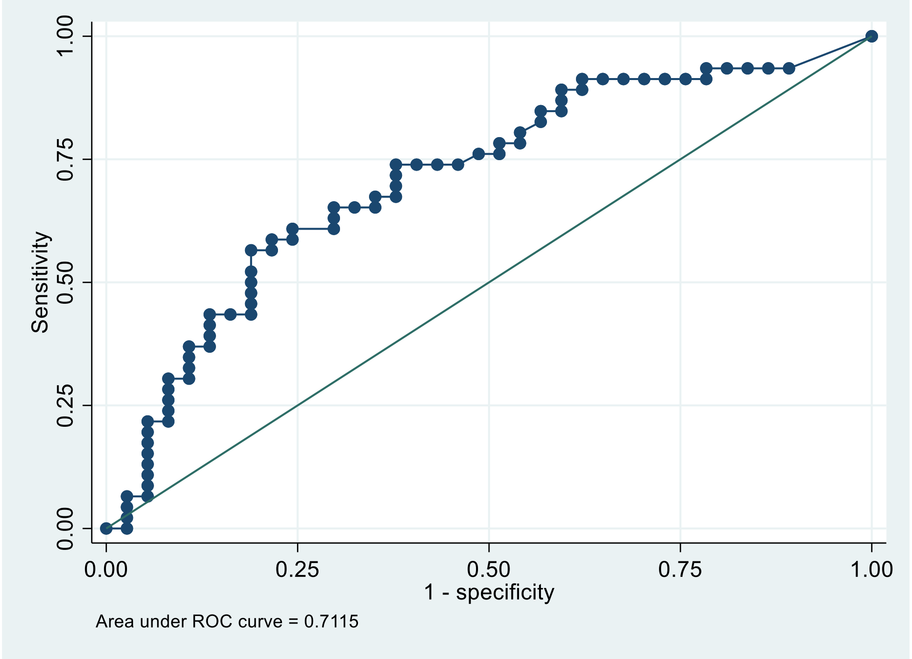

S. mansoni positives versus *S. mansoni* negatives

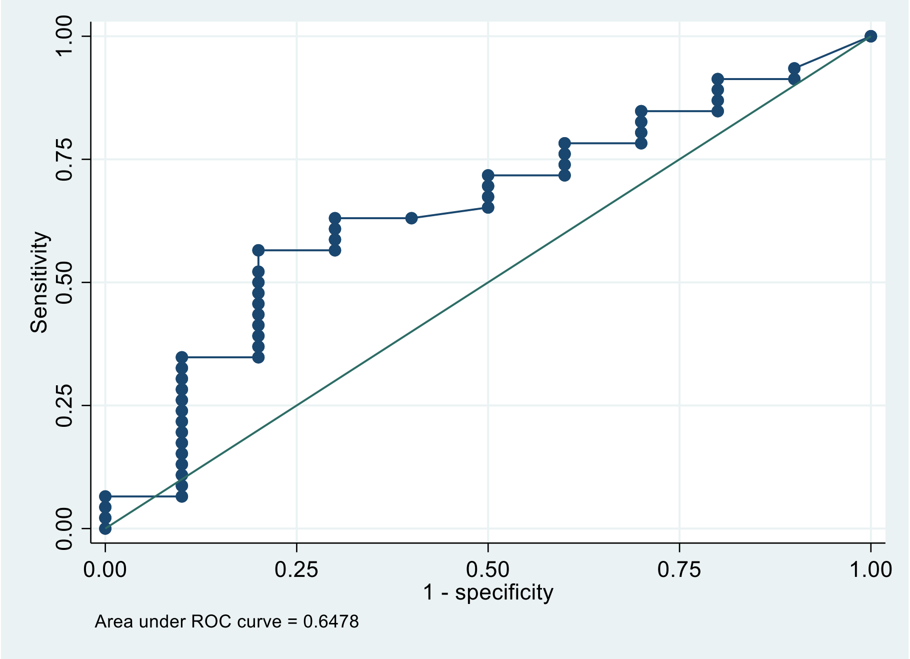

S. mansoni positives versus healthy controls

Peptide MS3_10186-25-41

m

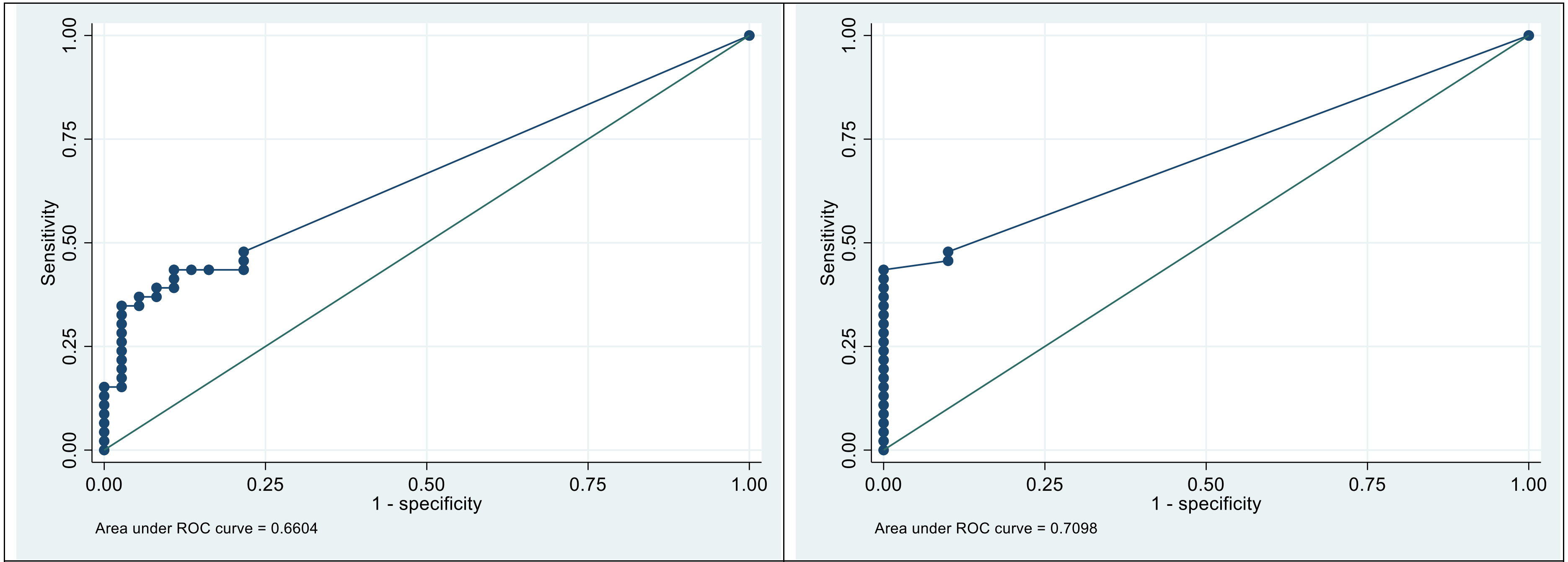

S. mansoni positives versus *S. mansoni* negatives	*S. mansoni* positives versus healthy controls

Peptide MS3_10186-40-49

n

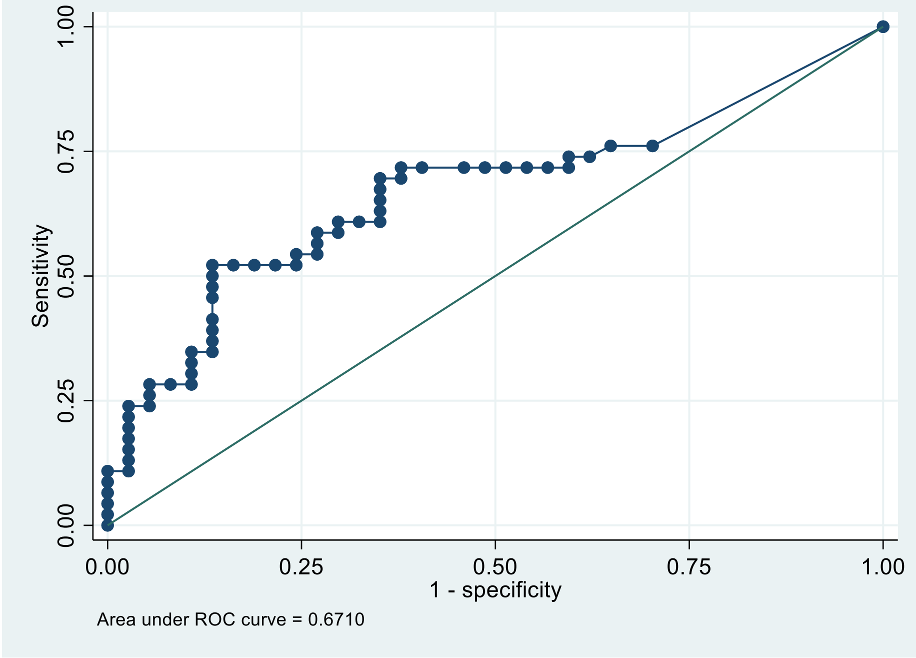

S. mansoni positives versus *S. mansoni* negatives

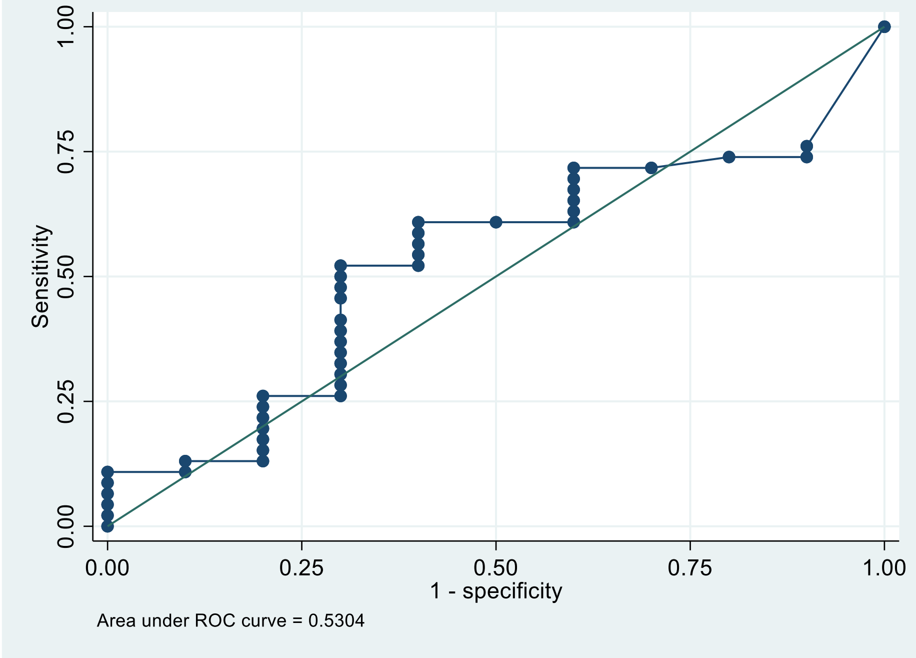

S. mansoni positives versus healthy controls

Peptide AAB81008-181-196

o

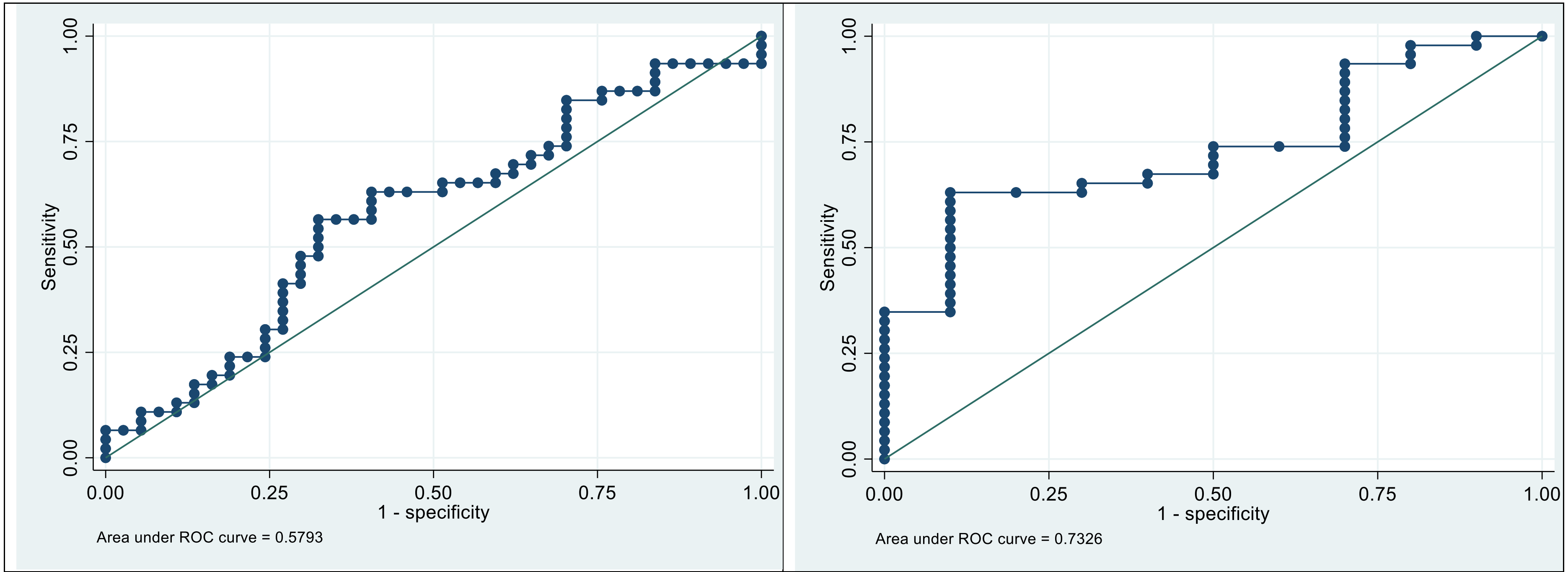

S. mansoni positives versus *S. mansoni* negatives	*S. mansoni* positives versus healthy controls
**Peptide SmSPI-197-214**	

p

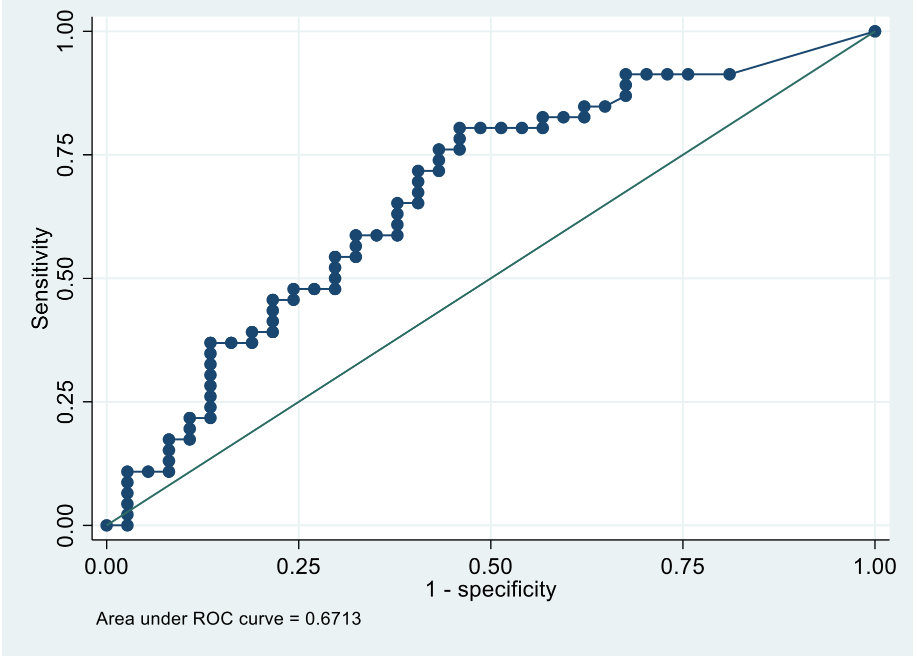

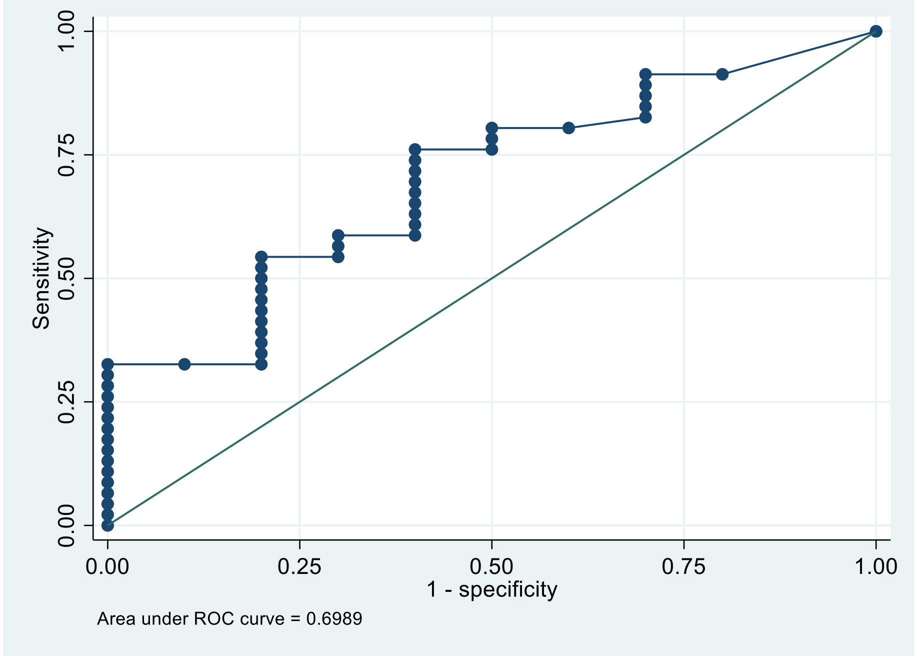

S. mansoni positives versus *S. mansoni* negatives	*S. mansoni* positives versus healthy controls
**Peptide Smp 150390.1-216-230**	

q

Diagnostic performance of peptides to detect *S. haematobium* patient IgG.

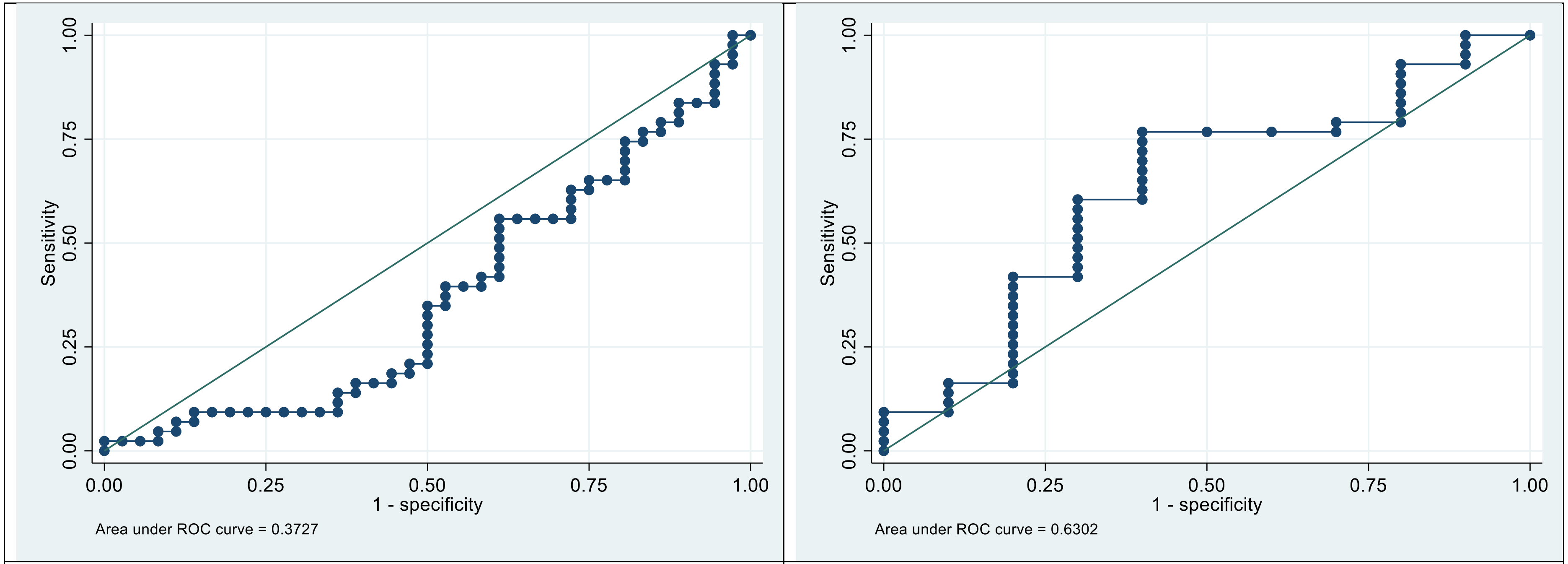

S. haematobium positives versus *S. haematobium* negatives	*S. haematobium* positives versus healthy controls
**Peptide SmSPI-177-193**	

r

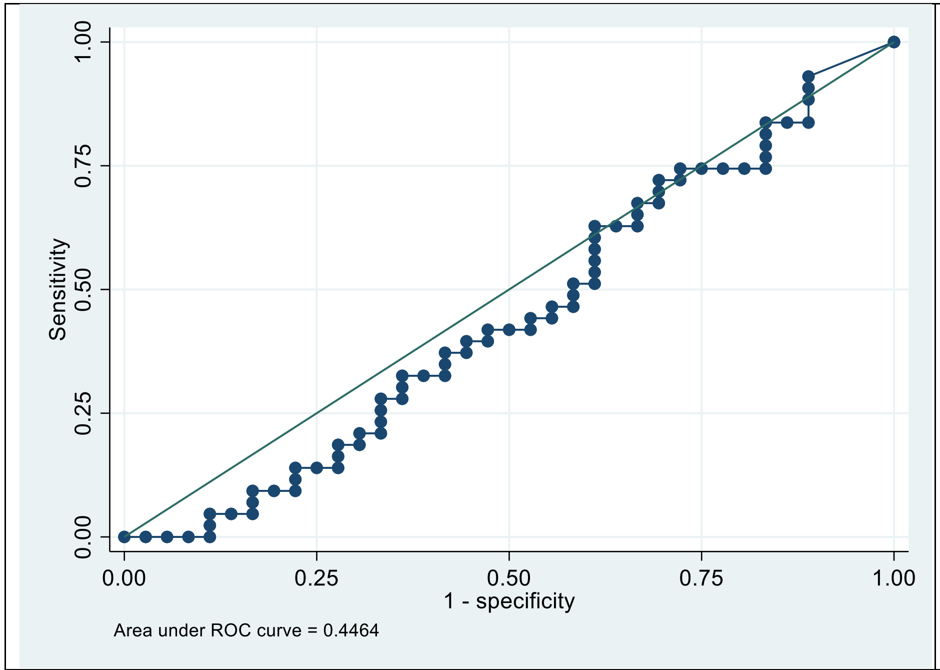

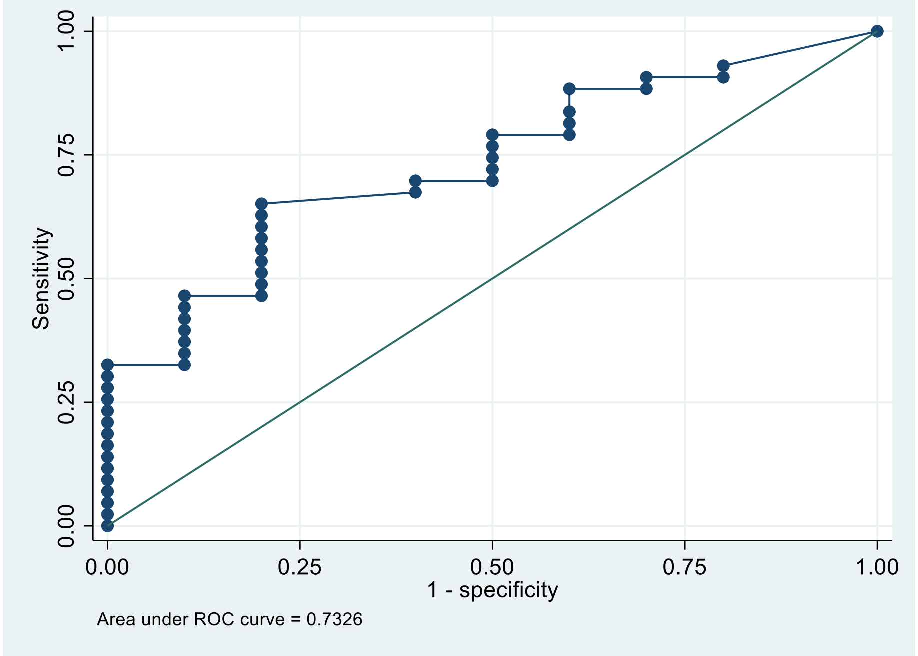

S. haematobium positives versus *S. haematobium* negatives	*S. haematobium* positives versus healthy controls

Peptide AAB81008-19-30

s

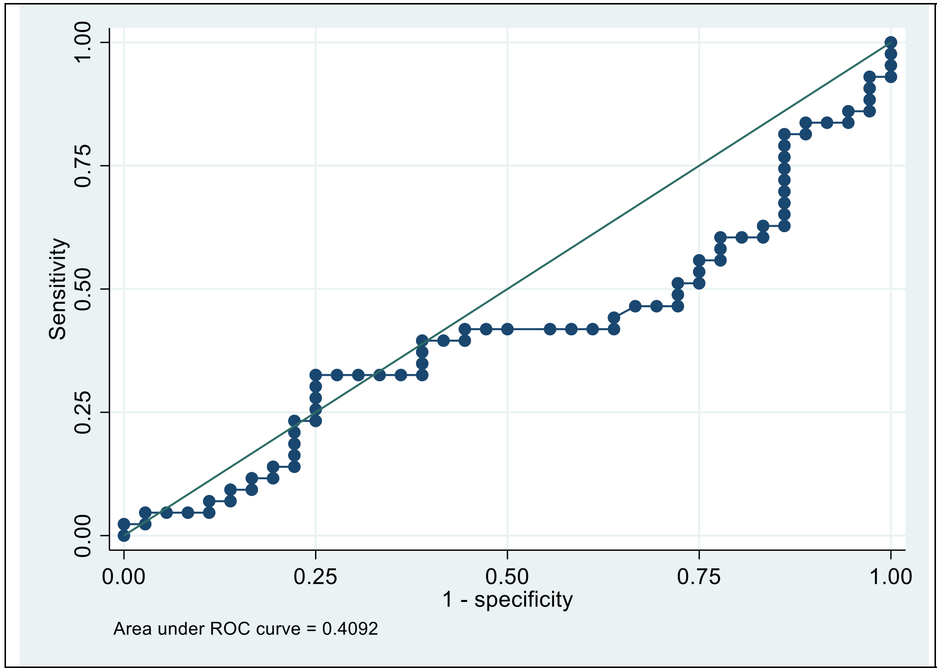

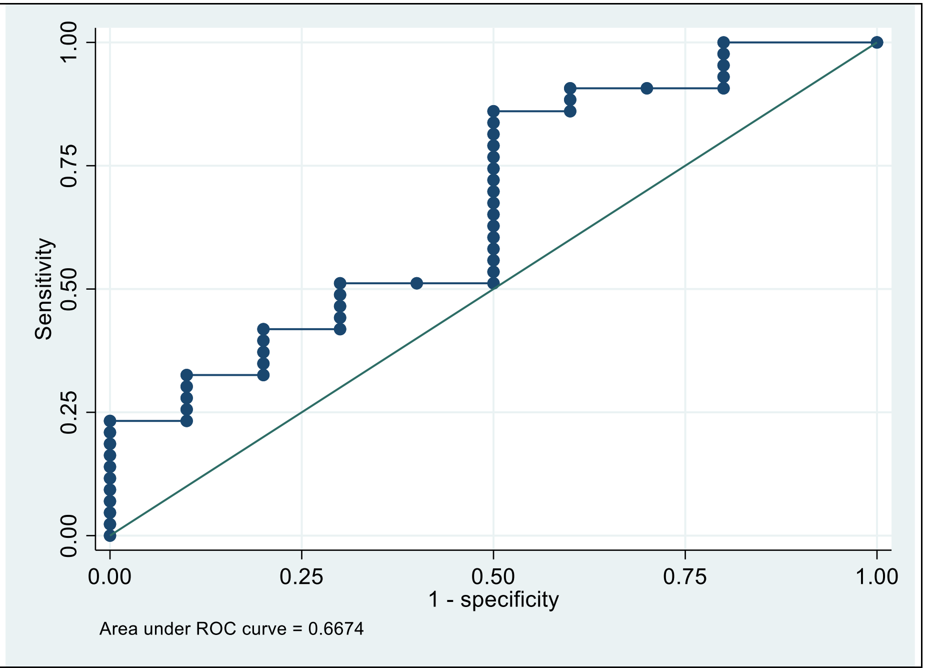

S. haematobium positives versus *S. haematobium* negatives	*S. haematobium* positives versus healthy controls
**Peptide SmSP1-165-181**	

t
