# Supplementary material for: Identification of Schistosoma haematobium and Schistosoma mansoni linear B-cell epitopes with diagnostic potential using in silico immunoinformatic tools and peptide microarray technology
Source: PLoS Negl Trop Dis. 2024 Aug 22;18(8):e0011887. doi: 10.1371/journal.pntd.0011887 (PMC11373837; doi:10.1371/journal.pntd.0011887)
Supplement: S6 File — Receiver operating characteristics (ROC) curve and area under the ROC curve (AUC) to detect S. mansoni (a-b) and S. haematobium (c) patient serum IgM. (PDF) [file pntd.0011887.s006.pdf]

Diagnostic performance of peptides to detect *S. mansoni* patient IgM.

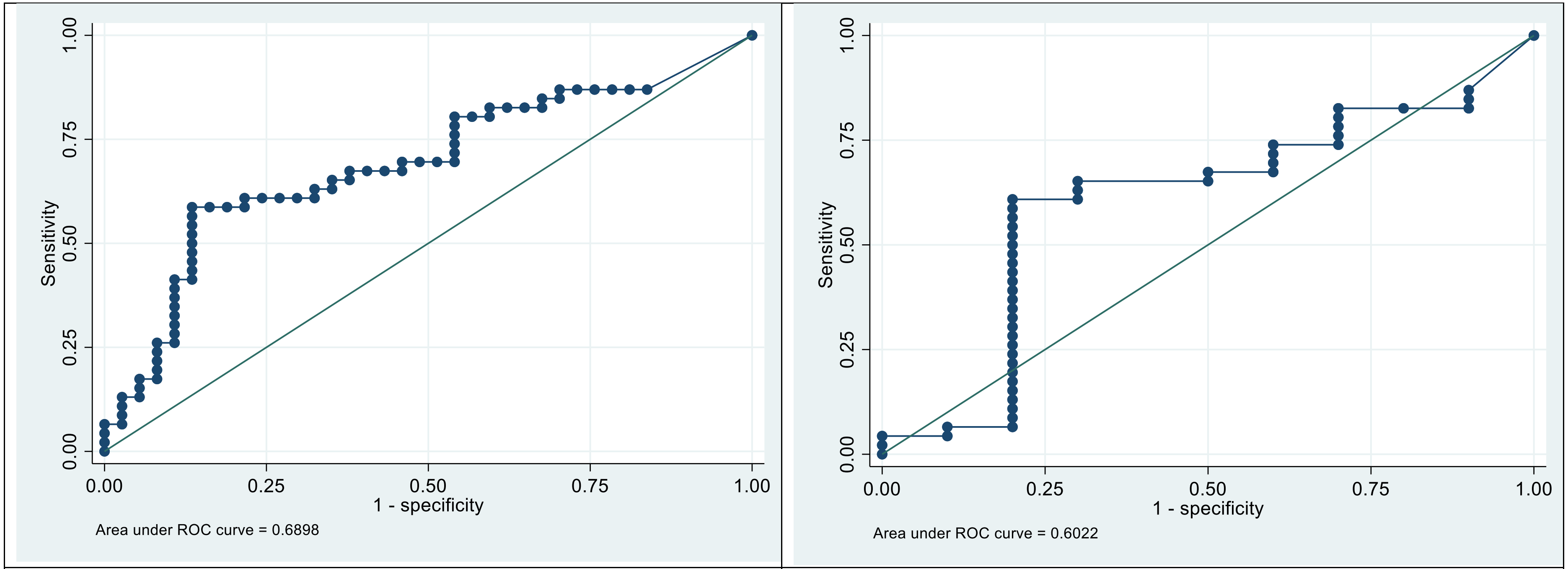

S. mansoni positives versus *S. mansoni* negatives	*S. mansoni* positive versus healthy controls
**Peptide MS3_10186-105-121**	

a

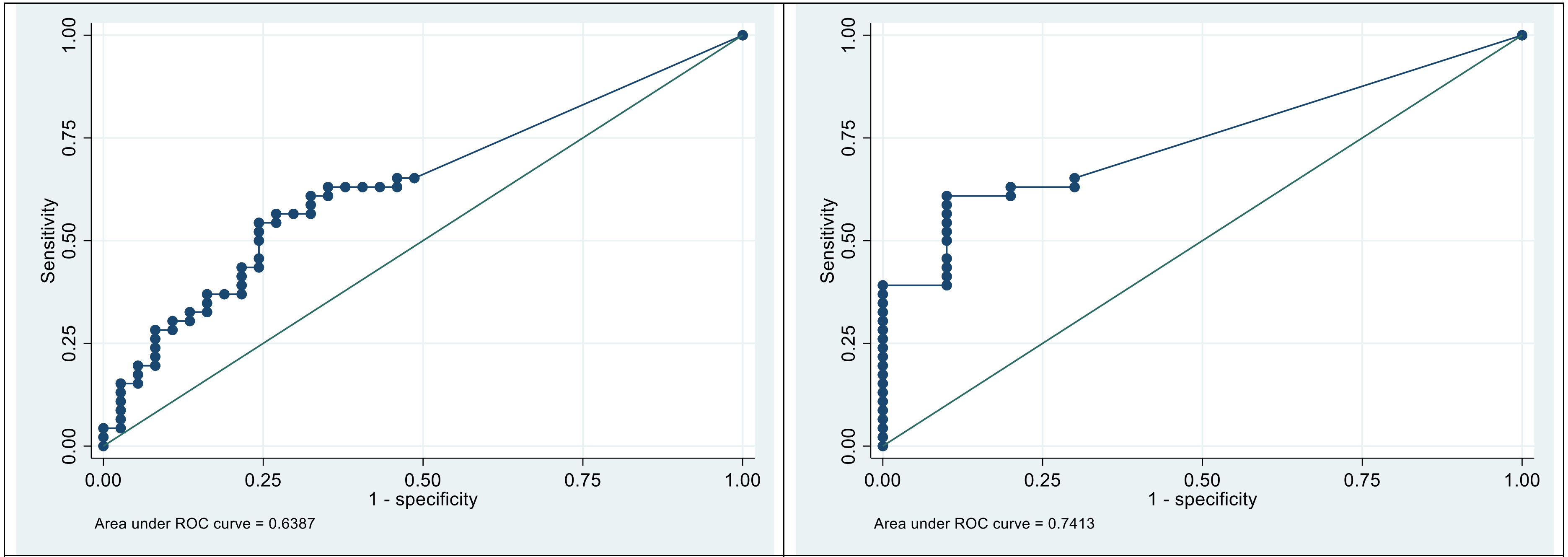

S. mansoni positives versus *S. mansoni* negatives	*S. mansoni* positive versus healthy controls

Peptide MS3_10186-40-49

b

Diagnostic performance of peptides to detect *S. haematobium* and patient IgM.

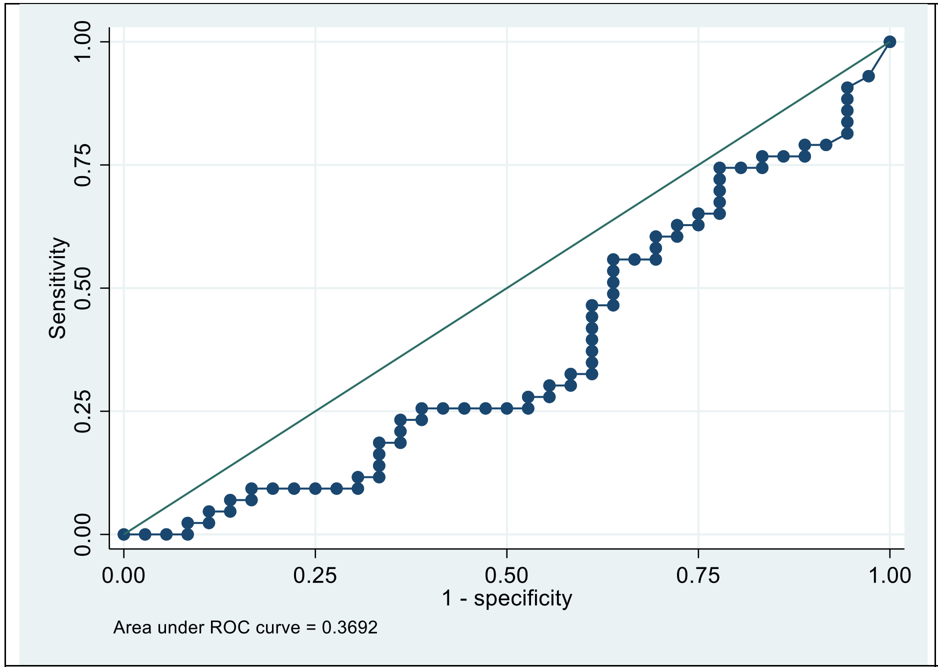

S. haematobium positives versus *S. haematobium* negatives

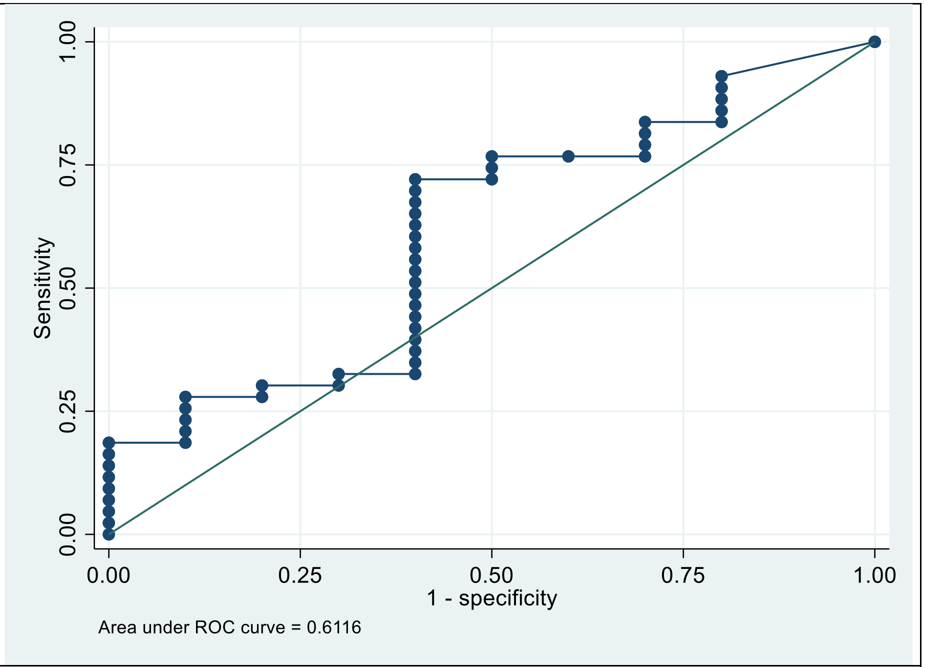

S. haematobium positive versus healthy controls

Peptide AAB81008-19-30

c
